# MDCT-based longitudinal automated airway and air trapping analysis in school-age children with mild cystic fibrosis lung disease

**DOI:** 10.3389/fped.2023.1068103

**Published:** 2023-02-02

**Authors:** Oliver Weinheimer, Philip Konietzke, Willi L. Wagner, Dorothea Weber, Beverly Newman, Craig J. Galbán, Hans-Ulrich Kauczor, Marcus A. Mall, Terry E. Robinson, Mark O. Wielpütz

**Affiliations:** ^1^ Department of Diagnostic and Interventional Radiology, University of Heidelberg, Heidelberg, Germany; ^2^ Translational Lung Research Center (TLRC), German Lung Research Center (DZL), University of Heidelberg, Heidelberg, Germany; ^3^ Department of Diagnostic and Interventional Radiology with Nuclear Medicine, Thoraxklinik at University of Heidelberg, Heidelberg, Germany; ^4^ Institute of Medical Biometry and Informatics (IMBI), University of Heidelberg, Heidelberg, Germany; ^5^ Department of Radiology, Stanford University School of Medicine, Stanford, CA, United States; ^6^ Department of Radiology, University of Michigan, Ann Arbor, United States; ^7^ Department of Pediatric Pulmonology, Immunology and Intensive Care Medicine, Charité-Universitätsmedizin Berlin, Berlin, Germany; ^8^ Berlin Institute of Health @ Charité-Universitätsmedizin Berlin, Berlin, Germany; ^9^ German Center for Lung Research (DZL), Associated Partner Site, Berlin, Germany; ^10^ Department of Pediatrics, Center of Excellence in Pulmonary Biology, Stanford University School of Medicine, Stanford, CA, United States

**Keywords:** Quantitative computer tomography, Bronchiectasis, Air trapping, Cystic Fibrosis, Automated analysis

## Abstract

**Objectives:**

Quantitative computed tomography (QCT) offers some promising markers to quantify cystic fibrosis (CF)-lung disease. Air trapping may precede irreversible bronchiectasis; therefore, the temporal interdependencies of functional and structural lung disease need to be further investigated. We aim to quantify airway dimensions and air trapping on chest CT of school-age children with mild CF-lung disease over two years.

**Methods:**

Fully-automatic software analyzed 144 serial spirometer-controlled chest CT scans of 36 children (median 12.1 (10.2–13.8) years) with mild CF-lung disease (median ppFEV1 98.5 (90.8–103.3) %) at baseline, 3, 12 and 24 months. The airway wall percentage (WP_5–10_), bronchiectasis index (BEI), as well as severe air trapping (A3) were calculated for the total lung and separately for all lobes. Mixed linear models were calculated, considering the lobar distribution of WP_5–10_, BEI and A3 cross-sectionally and longitudinally.

**Results:**

WP_5–10_ remained stable (*P* = 0.248), and BEI changed from 0.41 (0.28–0.7) to 0.54 (0.36–0.88) (*P* = 0.156) and A3 from 2.26% to 4.35% (*P* = 0.086) showing variability over two years. ppFEV1 was also stable (*P* = 0.276). A robust mixed linear model showed a cross-sectional, regional association between WP_5–10_ and A3 at each timepoint (*P* < 0.001). Further, BEI showed no cross-sectional, but another mixed model showed short-term longitudinal interdependencies with air trapping (*P* = 0.003).

**Conclusions:**

Robust linear/beta mixed models can still reveal interdependencies in medical data with high variability that remain hidden with simpler statistical methods. We could demonstrate cross-sectional, regional interdependencies between wall thickening and air trapping. Further, we show short-term regional interdependencies between air trapping and an increase in bronchiectasis. The data indicate that regional air trapping may precede the development of bronchiectasis. Quantitative CT may capture subtle disease progression and identify regional and temporal interdependencies of distinct manifestations of CF-lung disease.

## Introduction

Cystic fibrosis (CF) is a common life-limiting autosomal recessive genetic disorder, and morbidity and mortality are caused mainly by bronchiectasis, small airway obstruction, and progressive respiratory impairment (1). Spirometry and the lung clearance index (LCI) are important markers of disease severity and prognosis, delivering quantitative markers on patient condition (2). However, high-resolution chest CT is fast, accurate, highly available and radiation exposure continues to go down with newer generations of devices. It is more sensitive than spirometry for showing long-term disease progression, can more easily be performed in younger children. LCI is a rather time consuming technique and cannot replace CT for bronchiectasis screening (3, 4). Furthermore, CT offers promising markers (5–10) for the objective and automated measurement of functional and actual structural lung changes. Airway wall thickening and mucus plugging are early, potentially reversible airway changes thought to be linked to the development of bronchiectasis (11–14). Bronchiectasis develops early in the course of CF, being detectable in infants as young as 10 weeks of age, and is persistent and progressive (15). Functional and early structural lung disease are frequently observed in newborns and infants by signs of air trapping in CT, even in the absence of respiratory symptoms (15–17). Air trapping may precede structural lung damage, but the temporal and regional interdependencies of functional and structural lung disease have not been well studied on imaging (15). Furthermore, only limited data is available on the longitudinal changes of air trapping (8, 18), whereas the quantitative information on airway changes is mainly limited to cross-sectional observations (19).

The present study was conducted on 36 school-age CF subjects with a mild disease course to study QCT parameters on four consecutive time points over two years to address the following issues: (1) longitudinal development of airway parameters, bronchiectasis and air trapping over two years, (2) cross-sectional interdependencies between airway parameters, bronchiectasis and air trapping on a lobar level, and (3) longitudinal interdependencies of airway parameters, bronchiectasis, and air trapping over two years.

## Materials and methods

### Subjects

36 school-age children with CF underwent serial chest CT scans and lung function testing at baseline, 3, 12, and 24 months. The subjects were tested as part of a joint Novartis Pharmaceutical - Cystic Fibrosis Therapeutics Development Network Consortium study in 2007–2011 before the availability of cystic fibrosis transmembrane conductance regulator (CFTR) modulator therapy, evaluating the natural progression of lung disease. The subjects were diagnosed with mild CF lung disease defined at enrollment and baseline testing (% predicted forced vital capacity (ppFVC) >80%, forced expiratory volume in 1 s (ppFEV1) ≥75%, Table 1) (8). This study was conducted before the widespread use of the lung clearance index as a clinically useful lung function outcome measure in CF, which was therefore not included. Study subjects were clinically stable at testing day and had not received neither oral nor intravenous antibiotics for a minimum of 28 days before the study. Full details of recruitment, inclusion and exclusion criteria and institutional review board approval declaration are summarized in the online supplement.

**Table 1:** **Patient demographics**. Patient demographics (body mass index (BMI)) and spirometric data (forced vital capacity (FVC), forced expiratory volume in 1s (FEV1) and, forced expiratory flow at 25 and 75% of the lung volume (FEF25%–75%) are given at baseline, 3 months, 12 months, and 24 months. Percentage values refer to predicted volumes. Infection status for PA (Pseudomonas aeruginosa) and MRSA (chronic methicillin-resistant Staphylococcus aureus) is also shown.

	Baseline	3 months	12 months	24 months	*P*-value
Number of subjects	36				
Male/female	16/20				
Sweat Cl Value	100 (90.8–109.3)				
Homozygous F508del	23				
Age (y)	12.1 (10.2–13.8)	12.3 (10.4–14)	13.1 (11.2–14.8)	14.1 (12.3–15.8)	<0.001
Weight (kg)	41.25 (31.2–49.5)	42.6 (32.25–50.2)	45.7 (35–52.5)	47.9 (41.4–57.8)	<0.001
Height (cm)	147.5 (137.4–154.5)	150.4 (138.5–156)	151.7 (141.8–159)	155.5 (146.6–162)	<0.001
BMI (kg/m^2^)	18.4 (17.1–20.2)	18.7 (17.6–20.3)	19.3 (17.6–20.6)	19.9 (18.3–21.3)	<0.001
BMI (%*P*)	54.2 (40.2–73.9)	59.6 (41.2–73.6)	63.4 (44–81)	75.3 (58.3–84)	<0.001
FVC (l)	2.75 (2.1–3.2)	2.8 (2.1–3.4)	2.9 (2.4–3.7)	3.3 (2.7–4)	<0.001
ppFVC	99.9 (93.7–106.4)	100.3 (92.5–105.6)	98 (90.7–106.6)	104.5 (94.1–110.7)	0.506
FEV1 (l/s)	2.3 (1.8–2.8)	2.3 (1.7–2.9)	2.4 (1.9–3)	2.7 (2.1–3.2)	<0.001
ppFEV1	98.5 (90.8–103.3)	94.3 (83.9–103.3)	94.3 (86.3–101.5)	97.7 (90.5–102.9)	0.276
FEF25-75 (l/s)	2.6 (1.8–3.4)	2.6 (1.8–3.4)	2.6 (2–3.6)	2.8 (2–3.7)	0.015
ppFEF25%–75%	90 (77.5–109.1)	89.2 (63.1–104.3)	82.5 (66.9–102.7)	90.2 (67–97.9)	0.092
PA negative PA positive	31 (86.1%) 5 (13.9%)	30 (83.3%) 6 (16.7%)	29 (80.6%) 7 (19.4%)	28 (77.8%) 8 (22.2%)	0.714
MRSA negative MRSA positive	27 (75.0%) 9 (25.0%)	31 (86.1%) 5 (13.9%)	30 (83.3%) 6 (16.7%)	28 (77.8%) 8 (22.2%)	0.456

### Spirometry

Spirometry was obtained in the standing position for pulmonary function measurements. Pulmonary function measurements (FVC, FEV1, and FEF_25%–75%_) were expressed as percent predicted based on normal prediction equations derived from the Global Lung Function Initiative (GLI-2012) predictive equations for spirometric measurements subsequently generated from the ERS Global Lung function task force (20).

### Computed tomography

Chest CT scans and pulmonary function testing were obtained on the same day. Chest CT scans were obtained with spirometer-controlled acquisition using a spiral CT scanner at two CF centers. A low-dose volumetric CT protocol was utilized with paired inspiratory and expiratory scans. Further details are provided within the online supplement.

### Image assessment

The fully automated software YACTA (version 2.8.5.36) segmented and analyzed the airway tree, lungs and individual lobes on inspiratory and expiratory CT images. User interaction or manual correction of the segmentations was not required. Segmentation results were visually inspected by a reader with more than 5 years in chest radiology.

Beside the total lung volume (TLV) and the residual lung volume (RLV), the following previously described air trapping parameters were calculated on the basis of the lung parenchyma segmentation automatically: (1) RVC_856–950_ which is defined as the relative volume change between the expiratory and inspiratory lung volumes with attenuation between −856 and −950 HU divided by the total lung volume without emphysema. The index ranges from −1.0 to 0, greater values (closer to zero) mean more air trapping (21). (2) E/I MLA which is the expiratory to inspiratory ratio of mean lung attenuation with a range from 0 to 1.0, greater values mean more air trapping (21). (3) A1-A3 which use three patient specific thresholds for the definition of air trapping. A1 represents defects on the basis of liberal criteria (mild air-trapping), while A3 represent defects on the basis of stringent criteria as published previously (severe air-trapping). The size of the defect areas is expressed as a percentage of the analysed lung parenchyma region (22).

**Figure 1:**
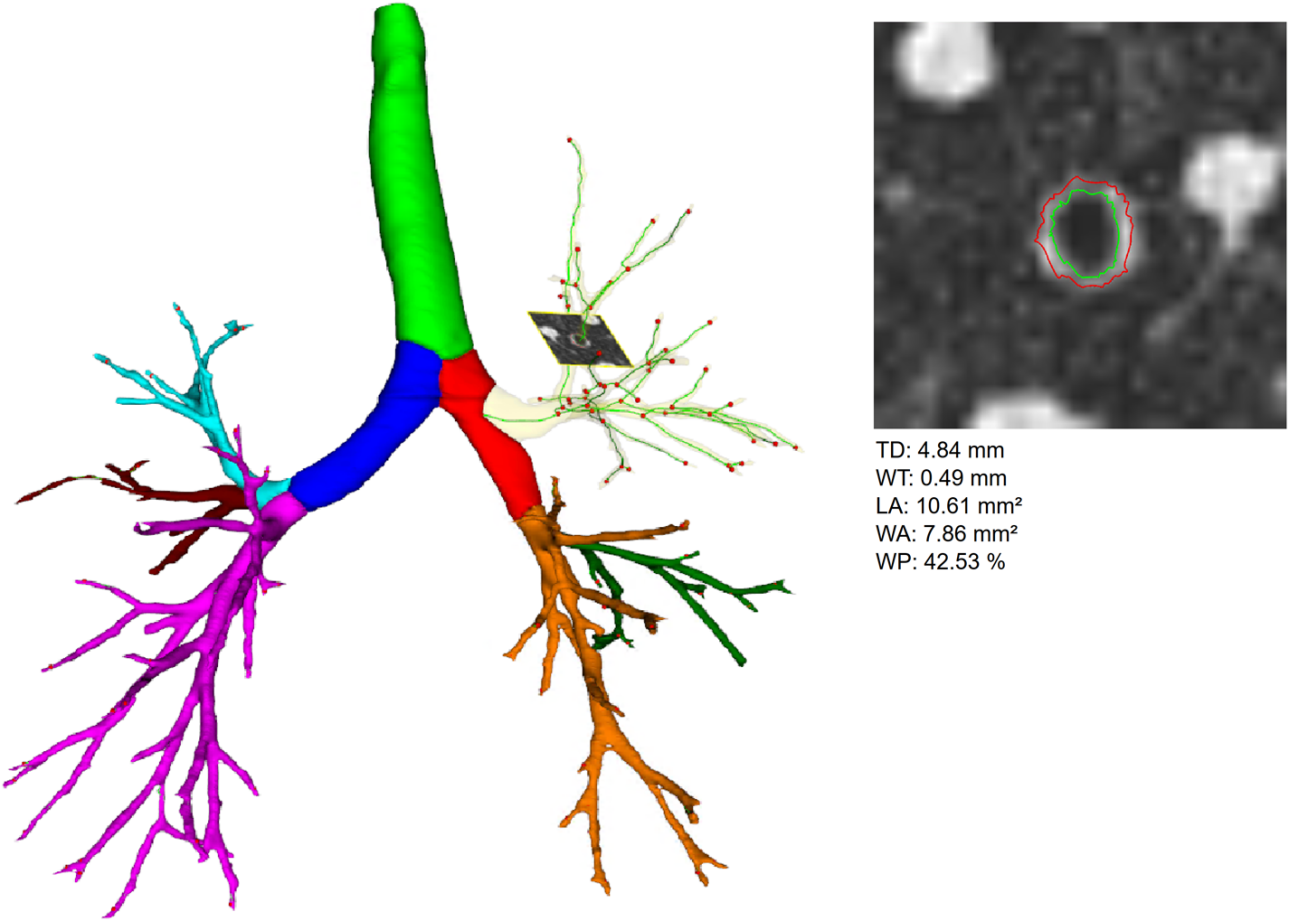
Left side shows a segmented airway tree including labeling of the pulmonary lobes, trachea highlighted in green, right main in red, left main in dark blue, right upper lobe in yellow (transparent), middle lobe in green and right lower lobe in orange, left upper lobe in light blue, lingula in dark red, and left lower lobe in pink. In the right upper lobe an orthogonal slice through an generation 6 airway is shown, on such slices, all airways of the whole segmented airway tree are measured. Right side shows the magnification of the orthogonal slice shown on the left side, inner (green) and outer (red) wall borders are displayed. Calculated airway parameters are given below. LA refers to the area within the green inner wall border. WA refers to the area between the green inner and red outer wall border. The other parameters are calculated from these values.

Segmentation of the airway tree was used to determine the following parameters automatically: (4) Bronchiectasis index (BEI) for the whole airway tree and for the individual lobes. The BEI calculation is based on the fact that the lumen of bronchi decreases with increasing generation number (airway tapering). Tapering of the airways is checked - if this is not the case, an error value is calculated, these error values are summed up for individual lobes or the entire lung. This approach is described in more detail in (7). (5) The basic geometry of the airways is described by the parameters total diameter (TD), wall thickness (WT), lumen area (LA), and wall percentage (WP = 100*WA/(LA + WA)) (Figure 1). These were determined generation-based in the trachea (G_1_), main stem (G_2_), lobar (G_3_), segmental (G_4_), and the aggregated subsegmental bronchi (G_5–__10_), the aggregated values were also calculated by lobe for G_5–10_. The primary location for airway disease in CF are usually the small airways defined as airways with an internal diameter below 2 mm, generally reflecting the 4th to the 14th generation of branching (23). Boon et al. made this observation by performing a morphometric analysis of explanted end-stage CF lungs, which showed extensive changes in the conducting airways, mainly from the 6th airway generation onward, describing dilatation and obstructions in up to 50% of the airways per generation (24). Therefore, we aggregated the airways generations 5th to 10th in our analysis, creating more resilient results due to the considerably higher amount of data and depicting the airway pathologies’ primary location.

Further details are provided within the online supplement.

### Statistical analysis

Results were summarized as the median and interquartile ranges for continuous variables and by absolute and relative frequencies for binary variables. Time-dependent changes in demographics, lung volume, air trapping, and airway parameters were assessed by Friedman Tests for continuous and by McNemar Tests for binary variables. The Wilcoxon-Mann-Whitney-*U*-Test is used to compare A3 values of patients with chronic infection with patients without. Pearson correlation coefficient was used for correlations and rated as suggested by Karlik (25). Robust linear/beta mixed models were calculated to study the regional relationship of airway structure and air trapping at single-timepoints. A second model was used to study the longitudinal interdependencies between airway changes and air trapping over two years. Since this is an explorative study, all *P*-values are descriptive. Further details are provided within the online supplement.

## Results

### School-age children with mild CF retain normal spirometry over two years

**Figure 2:**
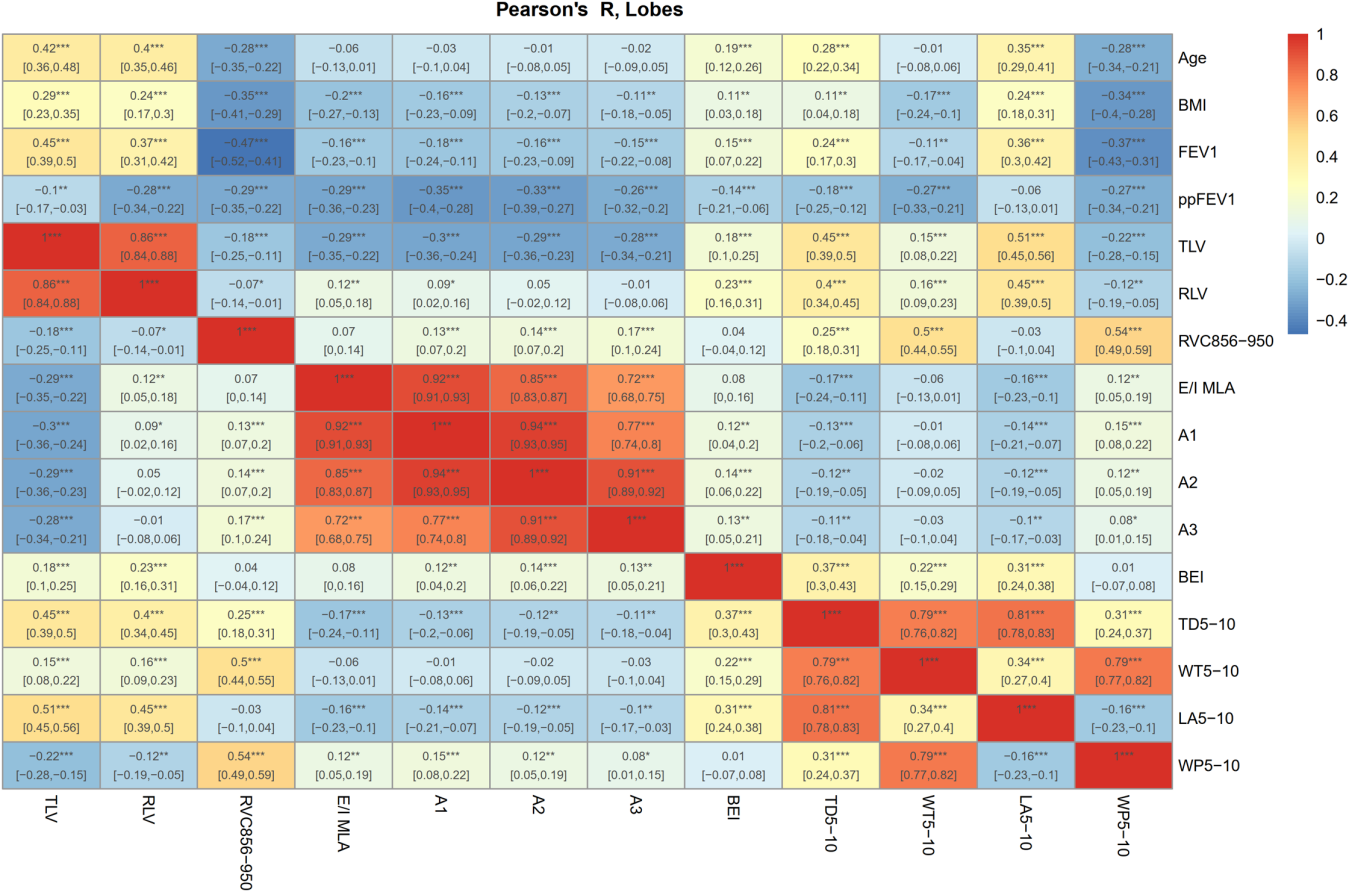
Correlation heat map of airway parameters and air trapping. Age, BMI, spirometric data FEV1 and ppFEV1, airway parameters (BEI, TD_5−10_, WT_5−10_, LA_5−10_, WP_5−10_), lung volumes (TLV, RLV) and air trapping parameters (RVC856-950, E/I MLA, A1-3) were correlated across all time points and lung lobes. Dark red shows strong positive correlations and dark blue shows strong negative. ***P < 0.001; **P < 0.01; *P < 0.05. 95% confidence interval in brackets. Benjamini-Hochberg method was used for adjustment for multiple testing (26). Correlation heat maps for the individual lobes are shown in Supplementary Figures S1A–G.

Patient demographics, such as weight, height, and BMI, increased substantially over two years (*P* < 0.001). Spirometric pulmonary function test values (FVC, FEV1, and FEF25%–75%) also increased significantly (*P* < 0.001), whereas predicted values (ppFVC, ppFEV1, and ppFEF25%–75%) did not deteriorate (*P* = 0.092–0.506) (Table 1). All airway parameters (BEI, TD_5–10_, WT_5–10_, LA_5–10_, WP_5–10_) were weakly (*r* = −0.06 to −0.27), and most of the air trapping parameters were moderately correlated with ppFEV1 (*r* = −0.26 to −0.35). All parameters were correlated across all time points and lung lobes. The lobe-based correlations showed no substantial differences (Figure 2 and Supplementary Figures S1A–G). The infection status regarding Pseudomonas aeruginosa (PA) and methicillin-resistant Staphylococcus aureus (MRSA) did not change considerably (Table 1 and Supplementary Table S1).

### Changing airway dimensions partially reflect patient growth

**Figure 3:**
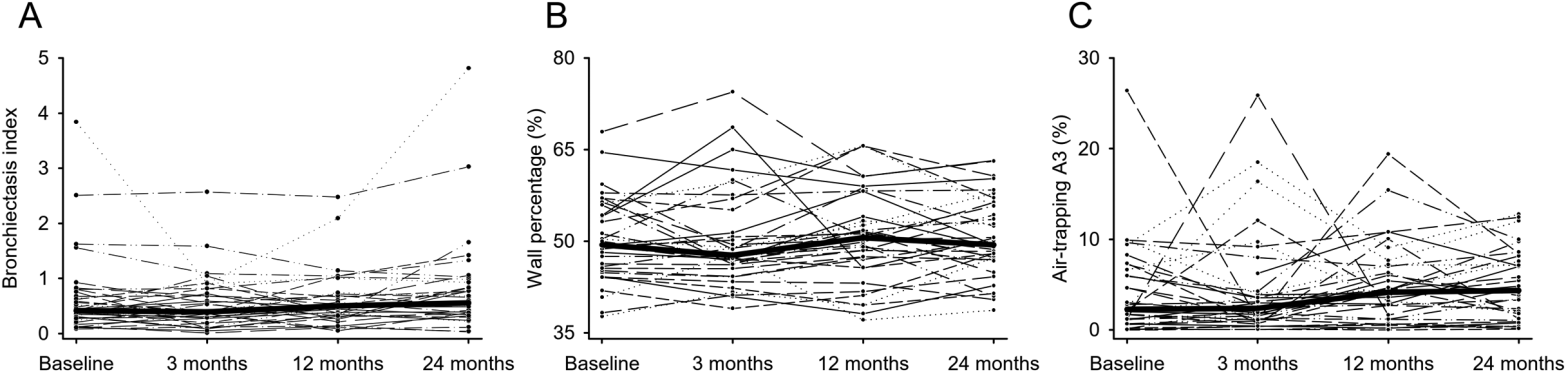
QCT measures over two years, the thin lines connect the values of the individual patients (*N* = 36) over time, the thick lines connect the median values. (**A**) The airway parameter bronchiectasis index (BEI) changed over two years from 0.41 to 0.54 (*P* = 0.156). (**B**) WP_5−10_ remained stable between baseline and 24 months (*P* = 0.248). (**C**) The air trapping parameter (A3) showed high variability at all time points, but increased the median A3 from 2.26% to 4.35% (*P* = 0.086).

**Figure 4:**
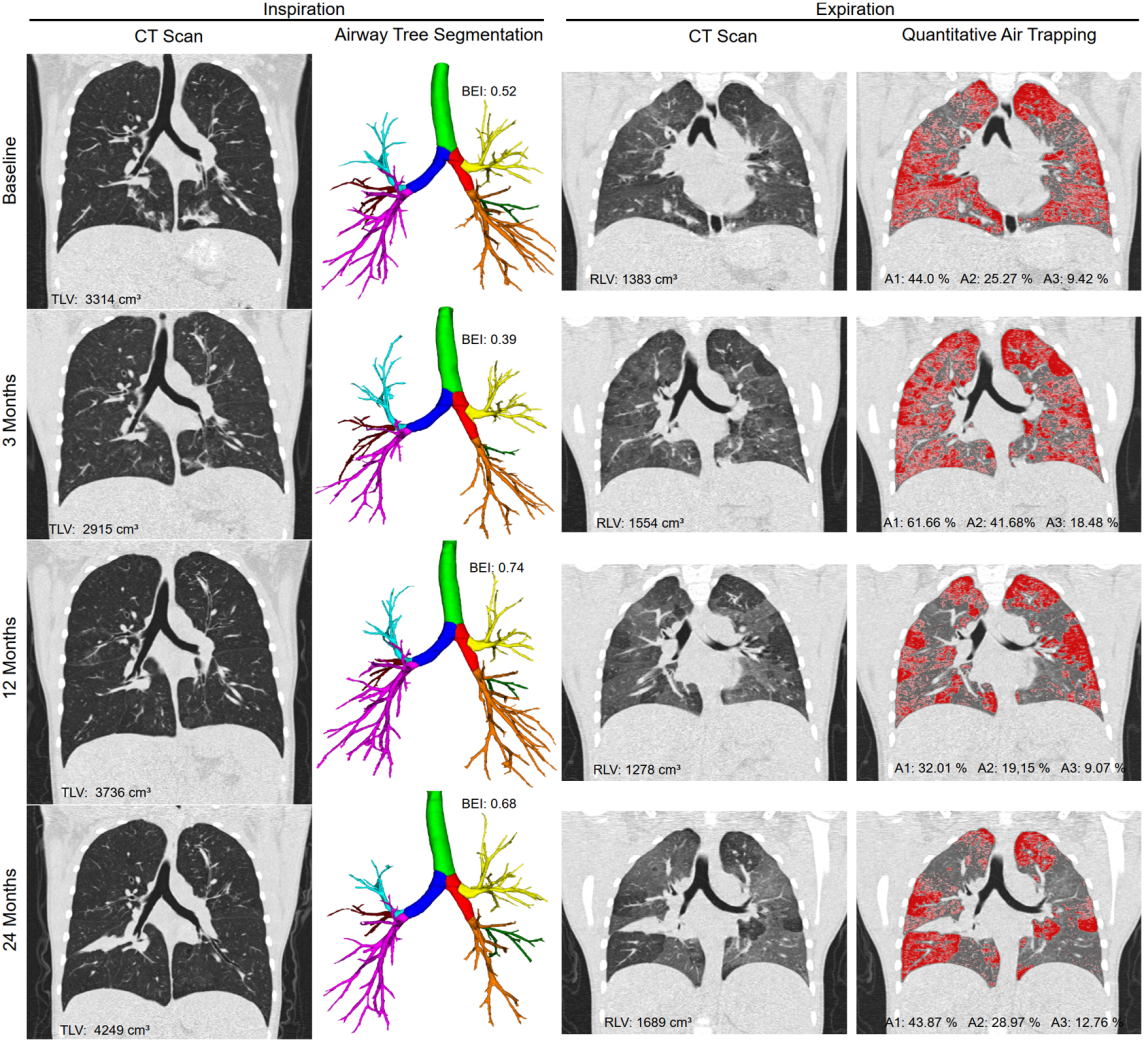
Representative example of a 10-year-old female cystic fibrosis patient scanned with paired inspiratory-expiratory CT at baseline, and subsequently after 3, 12 and 24 months. The first column shows the inspiratory CT in coronal reconstruction and the second column the segmentation results of the airway tree including labeling of the pulmonary lobes (trachea highlighted in green, right main in red, left main in dark blue, right upper lobe in yellow, middle lobe in green and right lower lobe in orange, left upper lobe in light blue, lingula in red, and left lower lobe in pink). The third column shows the expiratory CT in coronal reconstruction and the fourth column the results of air trapping quantification using the A1 (A1 includes A2 and A3) parameter. The quantitative results TLV, BEI, RLV, A1, A2, A3 are given for each timepoint. BEI increased from 0.52 to 0.68, and A3 from 9.42% to 12.76% over tow years. The corresponding ppFEV1 were 104% and 102%.

Longitudinal analysis of airway parameters and bronchiectasis was performed. For the total lung, the aggregated subsegmental airway parameters TD_5–10_, WT_5–10_, and LA_5–10_ increased (*P* < 0.05–0.001), whereas WP_5–10_ remained stable between baseline and 24 months (*P* = 0.248). The intra-individual course of airway parameters between time points showed substantial variability with a mean range of 12.47% for WP_5–10_ (Figure 3 and Table 2). BEI as a growth-independent measurement of disease severity changed over two years from 0.41 to 0.54 (*P* = 0.156). Airway tree segmentation and BEI values are shown for a representative example in Figure 4. Lobe-based airway analysis showed similar results for TD_5–10_ and LA_5–10_. BEI and WT_5–10_ increased in all lung lobes, except in the RML, where it decreased 0.16 to 0.12 and from 0.68 mm to 0.66 mm (*P* = 0.172, *P* = 0.540). WP_5–10_ showed a slight decrease in all lung lobes, except in the LLi, where it increased from 51.61% to 53.31% (*P* = 0.220). The intra-individual course also showed substantial variability in all lobes (Supplementary Figure S2 and Supplementary Table S2).

**Table 2:** **Temporal development of airway and air trapping parameters.** Airway parameters (BEI, TD, WT, LA, and WP), TLV, RLV and air trapping parameters (RVC856-950, E/I MLA, A1-3) were calculated for the whole lung at baseline, 3 months, 12 months, and 24 months. Airway parameters refer to the subsegmental airways generations 5th-10th.

Lung	Baseline	3 months	12 months	24 months	*P*-value
**Airways**					
BEI (−)	0.41 (0.28−0.7)	0.39 (0.29−0.77)	0.49 (0.32−0.7)	0.54 (0.36−0.88)	0.156
TD_5−10_ (mm)	5.04 (4.63−5.46)	4.96 (4.75−5.39)	5.15 (4.73−5.59)	5.35 (4.82−5.78)	**<0**.**001**
WT_5−10_ (mm)	0.77 (0.72−0.89)	0.78 (0.69−0.91)	0.84 (0.72−0.96)	0.84 (0.74−0.95)	**0**.**028**
LA_5−10_ (mm^2^)	9.99 (8.41−11.96)	10.21 (8.11−12.08)	10.9 (8.94−11.98)	11.36 (9.7−12.85)	**0**.**001**
WP_5−10_ (%)	49.41 (45.48−54.65)	47.69 (46.01−52.3)	50.59 (46.69−55.06)	49.41 (47.17−55.81)	0.248
**Parenchyma**					
TLV (cm^3^)	3740 (2986−4480)	3824 (3053−4549)	4052 (3249−4695)	4265 (3663−5217)	**<0**.**001**
RLV (cm³)	1262 (853−1505)	1165 (923−1558)	1336 (1003−1769)	1394 (1040−2086)	**<0**.**001**
RVC_856−950_	−0.63 (−0.69 to −0.53)	−0.62 (−0.68 to −0.54)	−0.62 (−0.71 to −0.47)	−0.63 (−0.7 to −0.53)	0.667
E/I MLA	0.64 (0.59−0.7)	0.66 (0.61−0.71)	0.68 (0.62−0.7)	0.67 (0.6−0.73)	0.982
A1 (%)	20.48 (11.78−37.9)	24.43 (14.39−34.99)	28.78 (18.59−38.04)	25.1 (14.97−42.08)	0.721
A2 (%)	8.8 (3.91−20.1)	11 (4.39−18.84)	14.05 (6.73−18.96)	12.96 (7.19−21.5)	0.257
A3 (%)	2.26 (1.11−6.47)	2.37 (1.13−4)	4.15 (1.91−6.24)	4.35 (1.84−7.47)	0.086

### Air trapping is variable over time

The longitudinal development of air trapping was analyzed. TLV and RLV increased over two years (*P* < 0.001), this also applies to all individual lobes. However, the air trapping parameters RVC_856–950_ and E/I MLA did not change substantially. The air trapping parameters A1-A3 showed the largest variation Where A1and A2 did not change significantly, A3 changed over two years from 2.26% to 4.35% (*P* = 0.086) (Table 2, Supplementary Table S3). However, the intra-individual course of air trapping parameters between time points shows substantial variability for the total lung with a mean range of 66.25% for A1, 47.34% for A2, and 21.05% for A3 (Figure 3). Air trapping segmentation and quantification values are shown for a representative examples in Figure 4 and Supplementary Figure S3. The individual lung lobes also showed differences in air trapping severity. The extent of severe air trapping (A3) at baseline varied, starting with 7.45% in the RML, compared to 0.88% in the RUL. Furthermore, the development of A3 was highly variable regarding individual lung lobes (Supplementary Figure S2 and Supplementary Table S3). The infection status of Pseudomonas aeruginosa (PA) and methicillin-resistant Staphylococcus aureus (MRSA) was not linked to changes in air trapping (Supplementary Table S1).

### Contribution of age to airway parameters and air trapping

Single-time point correlations between, age, airway changes and air trapping were performed using Pearson's correlation coefficient. The airway parameters TD_5–10_, LA_5–10_, and WP_5–10_ correlated weakly (*r* = 0.28, *r* = 0.35, *r *= –0.28), BEI very weakly (*r* = 0.19), and WT_5–10_ negligible with age (Figure 2). Lobe-based correlations showed no substantial differences (Supplementary Figures S1A–G). Age was moderately associated with an increase in TLV (*r* = 0.42). RVC_856–950_ showed a weak (*r* = −0.28), and the other air trapping parameters a very weak (*r* = −0.01 to –0.06) association with age.

On the total lung level, BEI had only very weak to weak correlations with other airway parameters (*r* = 0.01 to –0.37). The only moderate correlation was found between RVC_856–950_ and WT_5–10_, and WP_5–10_ (*r* = 0.5, *r* = 0.54) (Figure 2). The lobe-based correlations also showed no other associations between the severity of airway changes and the air trapping parameters (Supplementary Figures S1A–G).

### Wall percentage and air trapping showed cross-sectional interdependencies

A robust mixed linear model cross-correlated the airway and air trapping parameters of all individual lung lobes and all four examination time points to assess their regional relationship. The model pointed out that all parameters were linked to age (*P* = 0.003, *P* < 0.001, *P* = 0.014). Furthermore, WP_5–10_ was associated with the air trapping parameter A3 since an increase in A3 was linked to an increase in WP_5–10_ (*P* < 0.001). The examination time point also influenced WP_5–10_ (*P* = 0.021, *P* = 0.026, *P* = 0.036), whereas BEI and LA_5–10_ showed no dependency (Table 3).

**Table 3:** **Linear model to predict changes in airway parameters associated with air trapping.** Patient and lung regions are random effects. The fixed effects were age, A3, and the examination time point (3 months, 12 months, and 24 months), and the endpoints were the airways parameters BEI, LA_5−10_, and WP_5−10_. The regression coefficient (EstE) describes the influence of a fixed effect on the endpoints if it is increased by one unit. The confidence interval (Cl) with its lower and upper limits (LL – UL) and the standard error (SE) is also given.

	EstE Cl (LL – UL)	SE	*P*-value
**BEI**			
Age (y)	0.046 (0.018–0.073)	0.014	**0**.**003**
A3 (%)	0.003 (−0.002–0.008)	0.003	0.250
3 months	0.019 (−0.059–0.096)	0.039	0.639
12 months	0.015 (−0.068–0.098)	0.042	0.722
24 months	0.006 (−0.089–0.101)	0.049	0.902
**LA_5−10_**			
Age (y)	0.598 (0.407–0.789)	0.098	**<0**.**001**
A3 (%)	−0.006 (−0.034–0.021)	0.014	0.654
3 months	0.112 (−0.318–0.541)	0.219	0.614
12 months	−0.016 (−0.487–0.455)	0.24	0.947
24 months	−0.035 (−0.617–0.546)	0.297	0.906
**WP_5−10_**			
Age (y)	−0.042 (−0.07 to −0.014)	0.014	**0**.**014**
A3 (%)	0.004 (0.001–0.006)	0.001	**<0**.**001**
3 months	−0.025 (−0.067–0.016)	0.021	**0**.**021**
12 months	0.04 (−0.01–0.09)	0.026	**0**.**026**
24 months	0.086 (0.015–0.157)	0.036	**0**.**036**

### Bronchiectasis and air trapping showed short-term longitudinal interdependencies

A robust mixed linear model analyzed longitudinal interdependencies between airway and air trapping parameters in all lung lobes over two years. The model indicated that BEI was only affected by age between baseline and 3 months (*P* = 0.007), whereas LA_5–10_ was affected by age at all and WP_5–10_, inconsistently at some time intervals. Furthermore, a higher BEI, LA_5–10_, and WP_5–10_ at one examination time point seemed to be associated with a higher value at all following time points (*P* < 0.001). Lastly, BEI showed regional interdependencies with severe A3 over a short time interval of 3 months (*P* = 0.003) but not over longer time intervals of up to 2 years (*P* = 0.091) Table 4 and Supplementary Table S4).

**Table 4:** **Linear model to predict temporal development of airway parameters.** Patient and lung regions are random effects. The fixed effects were age, A3, and the examination time point (3 months, 12 months, and 24 months), and the endpoints were the airways parameters BE, LA_5−10_, and WP_5−10_. The regression coefficient (EstE) describes the influence on the fixed effect the change between one time point and the following time point. The confidence interval (Cl) with its lower and upper limits (LL – UL) and the standard error (SE) is also given.

	Est CI (LL – UL)	SE	*P*-value
**BEI**			
**Baseline and 3 months**			
Age (y)	0.043 (0.014–0.072)	0.015	**0**.**007**
A3 (%)	0.017 (0.007–0.028)	0.001	**0**.**003**
Baseline	0.403 (0.351–0.455)	0.027	**<0**.**001**
**Baseline and 24 months**			
Age (y)	0.230 (−0.002–0.048)	0.013	0.086
A3 (%)	0.008 (−0.001–0.013)	0.005	0.091
Baseline	0.983 (0.925–1.041)	0.030	**<0**.**001**
**LA_5−10_**			
**Baseline and 3 months**			
Age (y)	0.357 (0.139–0.575)	0.111	**0**.**003**
A3 (%)	0.023 (−0.031–0.077)	0.028	0.413
Baseline	0.378 (0.285–0.471)	0.048	**<0**.**001**
**Baseline and 24 months**			
Age (y)	0.293 (0.132–0.454)	0.082	**0**.**001**
A3 (%)	0.026 (−0.028–0.079)	0.027	0.356
Baseline	0.552 (0.443–0.660)	0.055	**<0**.**001**
**WP_5−10_**			
**Baseline and 3 months**			
Age (y)	−0.782 (−1.332– −0.233)	0.28	**0**.**009**
A3 (%)	0.013 (−0.088– 0.114)	0.052	0.796
Baseline	0.404 (0.292–0.517)	0.057	**<0**.**001**
**Baseline and 24 months**			
Age (y)	−0.992 (−1.466– −0.519)	0.242	**<0**.**001**
A3 (%)	0.1 (−0.021–0.221)	0.062	0.114
Baseline	0.386 (0.261–0.511)	0.064	**<0**.**001**

## Discussion

The present study was conducted on 36 school-age CF subjects with a mild disease course to give contemporary insight into the 2-year natural history of functional and structural lung disease in the absence of current CFTR modulatory therapy. We performed a longitudinal analysis of airway parameters for the total lung and all lung lobes as a first step. CF-lung disease is characterized by chronic airway inflammation and infection, leading to airway wall thickening and mucus plugging as early, potentially reversible airway changes (13). We used the airway parameters total diameter (TD_5–10_), lumen area (LA_5–10_), wall thickness (WT_5–10_), and wall percentage (WP_5–10_), which should reflect dilatations and obstructions equally well. We found a significant increase in TD_5–10_, WT_5–10_, and LA_5–10_ (*P* < 0.001–0.05), whereas the relative parameter WP_5–10_ remained stable between baseline and 24 months (*P* = 0.248). Heterogeneity is also a key feature of CF-lung disease. As expected, all airway parameters showed substantial variability within the patient cohort and between different lung lobes. For example, WP_5–10_ had an interquartile range of 45.48%–54.65% at baseline, with minimum and maximum values of 37.68% and 67.93%, respectively. A major obstacle when interpreting this data is separating the effects of growth and disease activity. However, we believe that WP_5–10_ as a ratio in which both the numerator and the denominator grow with age may be less affected by growth than the other parameters.

Bronchiectasis can be quantified using airway tapering, which should in turn be a growth-independent measure. Kuo et al*.* described the outer airway diameter to be reliable to diagnose bronchiectasis when performing visual scoring (10), but in our experience the inner airway diameter seemed more reliable (7). The BEI as a quantitative parameter has two advantages. Firstly, unlike the other airway parameters, BEI should be less variable and potentially less prone to changes during spontaneous improvements under therapy because bronchiectasis represents irreversible structural lung damage (18). Secondly, BEI can be considered less growth-dependent, and does not require the adjacent vessel to be compared with, which is another parameter introducing variability. Bronchiectasis are persistent and progressive, developing early in the course of CF and being detectable in infants as young as 10 weeks of age (15). In preschool children, the extent and severity of bronchiectasis is between 29.3%–61.5%, making the usefulness of a bronchiectasis score as an outcome measure for CF lung disease questionable (27, 28). However, in school-age children, bronchiectasis has a prevalence of 60% to 80%, making it a more and more relevant disease marker with increasing age. In our cohort, BEI changed over two years from 0.41 to 0.54 (*P* = 0.156), which was also in line with the literature (10). BEI also had a high regional heterogeneity with inconsistent progress in different lobes. We observed the highest increase in the RUL and RLL, which was partly in line with previous publications that observed more severe abnormalities and inflammation in the RUL (12, 16, 29). However, the highest BEI was found in the RLL. In summary, we showed that TD_5–10_, LA_5–10_, WT_5–10_, and WP_5–10_ most likely reflect a combination of growth and small airway disease with airway wall thickening and mucus plugging as early potentially reversible airway pathologies. The BEI, on the other hand, represents more irreversible structural lung damage. The lobe-based analysis also showed substantial variability between different lung lobes, again emphasizing the need for a quantitative assessment of regional disease activity.

Quantitative air trapping is a CT outcome measure that has promise in detecting early regional small and large airway obstruction before global lung function decline and progressive structural lung disease occurs. Currently, no specific air trapping parameter is considered a gold standard. Therefore, we tested the most commonly used air trapping parameters RV_C856–950_ and E/I MLA, which are normally used in adults, and the parameters A1-A3, which are adjusted for use in pediatric patients (22). RVC_856–950_ had a weak (*r* = −0.28) association with age and did not increase over two years. The use of fixed thresholds makes the parameter vulnerable to growth-related changes in lung density. This dependency might be especially problematic in the first years of life when lung density decreases linearly, approximating adult levels during adolescence (30, 31). On the other hand, E/I MLA and A1-A3 seemed not to be associated with age (*r* = −0.06; *r* = −0.01 to −0.03) since both parameters are less influenced by growth-related changes due to the way they are calculated. E/I MLA is the expiratory to inspiratory ratio of mean lung attenuation (21) and, A1-A3 is defined by using three dynamic thresholds (22), compensating for ages related changes in lung density. Accordingly, RVC_856–950_ correlated weakly with the other air trapping parameters (*r* = 0.07 to 0.17), whereas the E/I MLA and A1-A3 showed strong or very strong correlations with each other (*r* = 0.72 to 0.94). E/I MLA did not change substantially over two years, indicating that E/I MLA and RVC_856–950_ might not be sensitive enough to detect subtle changes in air trapping in school-age children. Therefore, we decided to focus on A1-A3. Indeed we found that A3 representing severe air trapping increased from 2.26% to 4.35% (*P* = 0.086), this parameter seems to be best suited for our patient collective.

Next, we analyzed cross-sectional interdependencies between airway parameters and air trapping at each timepoint to gain further insight into CF lung disease. Airway wall thickening and “trapped gas” behind closed airways showed associations in other lung diseases like bronchial asthma (32). We expected that the same associations exist in CF since airway inflammation also leads to airway wall thickening and airway obstruction. The assumption is strengthened by regional variations in wall percentage (WP_5–10_) and air trapping (A3), as observed in steps one and two. Furthermore, we also expected cross-sectional interdependencies between bronchiectasis and air trapping since in patients with CF, bronchiectasis are detected in 30% and air-trapping in 45% with CT (33). We believe that our study collective is predestined for showing connections between bronchiectasis and air trapping since only patients with mild CF-lung disease were included, making it likely to catch the development of irreversible lung changes at an early stage. Therefore, we applied a robust mixed linear model to consider the regional heterogeneity of CF by integrating lobe-based information to predict changes in BEI, LA_5–10_, and WP_5–10_ associated with age, air trapping, and the examination time point 3-month, 12-month, and 24-month. For our model we chose BEI, LA_5–10_, and WP_5–10_ as a reduced set of airway parameter input variables. The reasoning behind it was that pathological airway wall thickening might be best represented by WP_5–10_ since the parameter is less influenced by growth. Furthermore, all airway parameters had low correlations with ppFEV1, while WP_5–10_ was the most promising (*r* = −0.27). LA_5–10_ was chosen since the remaining parameters were strongly correlated with and are partly computationally derived from each other (e.g., TD - WT = LA).

Importantly, the model showed that A3 was significantly associated with higher WP_5–10_ (*P* < 0.001), implying that narrowing of peripheral airways and a thickening of the airway walls by mucus or inflammation is regionally connected to functional airway disease at a given timepoint. However, a higher A3 was not significantly associated with higher BEI (*P* = 0.250), indicating that bronchiectasis may not be cross-sectionally linked to regional air trapping at this stage of disease. As expected, BEI had a significant association with increasing age, representing irreversible bronchiectasis progression over time (*P* = 0.003). Therefore, especially structural changes of CF-lung disease like bronchiectasis might get better detected at 12- and 24-months. Age was also associated with higher LA_5–10_ (*P* < 0.001) and lower WP_5–10_ (*P* = 0.014). Higher LA_5–10_ was most likely attributed to growth and the lower WP_5–10_ with older age possibly reflects age-related structural changes of the airways (34, 35). However, this makes it more likely that the increasing WP_5–10_ in areas with air trapping are linked to pathology. The associations between LA_5–10_, WP_5–10_ and the study time points were inconsistent. The later time points, 12-months, and 24-months were associated with decreased LA_5–10_ and increased in WP_5–10_, while we found opposing tendencies at 3-month. This observation might be explained by the short observation period, where the high variability of CF-lung disease has a significantly greater influence than on longer observation periods.

Taken together, we could demonstrate a cross-sectional association between severe air trapping (A3) and WP_5–10_, indicating that measurable airway wall thickening is regionally linked to air trapping. Further, our model could not confirm a statistically significant regional association between air trapping and bronchiectasis.

Finally, we applied a second linear model to analyze the longitudinal interdependencies between airway changes and air trapping also by lobe over two years. Our model showed that age alone did not predict an increase in BEI, but higher BEI at baseline predicted higher BEI in the follow-up examinations. Furthermore, A3 was associated with higher BEI only between baseline and three months, indicating that more severe quantitative air trapping was predictive for developing bronchiectasis during the next three months. This observation suggested that there might be an association between BEI and A3 but only in a close temporal context but not over two years. Tepper et al*.* made the same observation in their study, finding no clearly identifiable pre-stages for the development of bronchiectasis in a two year timeframe (14). Therefore, our data might support the hypothesis made by Tepper et al., that the development of bronchiectasis is frequently an acute process and not caused by a slow continuous progressive transition, indicating that there are two possible phenotypes: one for rapid progression of bronchiectasis and one for more slowly developing bronchiectasis (14). The severity of A3 at baseline does not seem to predict the size of LA_5–10_ at 3-, 12-, and 24 months, while A3 at baseline was associated with an increase of WP_5–10_ at 12 months, but not for the other time points. We observed that higher values of the airway parameter LA_5–10_ at baseline independently predicts higher values of LA_5–10_ in all the following CT examinations, which might be attributable to growth since larger children at baseline will retain relatively larger airways follow-up exams. We observed the same for WP_5–10_ where higher values at baseline also independently predict higher WP_5–10_ at follow-up; however, age and WP_5–10_ were inconsistently associated. This implies that older children are more likely to have increased disease severity as measured by relative wall thickening in the following CT examinations, which partly matches with the observation by Mott et al*.* that wall thickening is not always reversible (17).

Collectively, we could strengthen the assumption that development of bronchiectasis might be an acute process by detecting regional interdependencies between severe air trapping and an increase in bronchiectasis over a short time interval of 3 months but not over longer time intervals of up to 2 years. However, the model failed to show a general dependency between bronchiectasis and air trapping which is consistent with previous findings that bronchiectasis can develop within two years without visible pre-stages (14). Therefore, bronchiectasis and air trapping are co-existent in many patients which was also reported by Boon et al*.,* who correlated multidetector computed tomography and micro-CT with thin section histology on explanted end-stage CF lungs, describing a wide variability between patients with predominance of large bronchiectasis with pronounced destruction in some patients, and hyperinflation with small airways obstruction in other patients (24).

Our study has some limitations. First, our study did not evaluate emerging physiologic lung function measures, such as the lung clearance index, which has also been shown to be a more sensitive measure than spirometry in CF clinical studies (36, 37). In a study in 60 school-aged children, LCI and CT scoring had similar sensitivity to detect CF lung disease, and in some instances, both provided complementary information for detection of the underlying disease (36). Second, in most cases of our cohort, we assume that a stable full inspiratory and end-expiratory breath-hold was achieved due to spirometer-controlled CT. This assumption is supported by a mean lung volume change <5% between single acquisitions, which is also less than the 10% recommended by Madani et al. (38). But it is obvious that the level of inspiration and expiration certainly influences the presented QCT results. As expected, the total lung volume (TLV) was moderately associated with age in our patient cohort, increasing by approximately 14% over two years. Some authors normalize for lung volume since quantitative lung parameters, and airway dimensions depend on height, body weight, and gender (39–41). Nevertheless, we waived normalization because we believe it may have confounded our results. The majority of studies normalizing for lung volume included adults with mature lungs, whereas growth is an inherent factor in school-age children. Growth also influences the number of airways detected by CT since chest CT resolution is an essential determinant for the smallest structures that can still be observed. CT measurements are consistently accurate and reproducible in airway diameters down to approximately 2 mm (42, 43), meaning that small intrapulmonary airways of higher generations are below the resolution limit. Therefore, it seems possible that the number of detectable airways is also affected by the size of the patient, which might influence airways analysis in a patient cohort with growing lungs. Third, mucus plugging was identified as an indicator for bronchiectasis 6 years later and as a potential pre-stage of bronchiectasis (14, 44). In our study mucus plugging is only indirectly represented by LA_5–10_ and also WP_5–10_ in cases where a mucus layer covers the airway surface without complete obstruction, then LA_5–10_ decreases and WP_5–10_ increases. If an airway is completely closed by mucus, airway segmentation fails and no airway parameters can be determined at all. To our knowledge, no QCT marker for mucus plugging is available.

In summary, we could demonstrate that QCT is feasible to detected subtle airway and parenchymal changes in school-age children with mild CF lung disease. Despite the difficulty of distinguishing growth from disease-related changes, we detected a trend to increasing severe air-trapping and bronchiectasis over two years. By fitting the regional and temporal information of CF-related changes into two mixed linear models, we could demonstrate cross-sectional, regional interdependencies between wall thickening and severe air trapping. Furthermore, we could detect regional interdependencies between severe air trapping and an increase in bronchiectasis over a short time interval of 3 months but not over longer time intervals of up to 2 years, indicating that air trapping might precede the development of bronchiectasis as an acute process. Therefore, we believe that disease quantification with QCT should include airway parameters and air trapping, since both are important and only partially linked markers for disease severity.

We were able to show that automated evaluation offers distinct advantages in providing fast, efficient, reproducible and comparable detailed longitudinal data, much more difficult with direct human observation resp. scoring. Knowledge of the natural course of the disease is important in order to better understand and assess the effect of current CFTR modulatory therapy or other new drugs.

## Data Availability

All relevant data are within the manuscript and its Supporting Information files.
