# Supplementary material for: MDCT-based longitudinal automated airway and air trapping analysis in school-age children with mild cystic fibrosis lung disease
Source: Front Pediatr. 2023 Feb 2;11:1068103. doi: 10.3389/fped.2023.1068103 (PMC9932328; doi:10.3389/fped.2023.1068103)
Supplement: Supplementary file 1 [file Table1.docx]

Supplementary Material

# Supplementary Data

## Subjects

Thirty-seven school-age children with mild CF lung disease were prospectively enrolled as part of a joint Novartis Pharmaceutical - Cystic Fibrosis Therapeutics Development Network Consortium study evaluating the natural progression of lung disease in children with mild CF defined at enrollment and baseline testing. Qualifying subjects were accrued from Lucile Packard Children’s Hospital (Palo Alto, CA) and Nationwide Children’s Hospital (Columbus, Ohio) and were followed for two-years from 2007 - 2011. All subjects had a confirmed CF diagnosis based on a pilocarpine iontophoresis sweat chloride testing (≥ 60 mEq/L) and a genetic analysis for Class I – III CFTR gene mutations. This study’s inclusion criteria included routine medical care in the CF clinic, age 7 to 18 years, percent predicted FVC ≥ 85 %, a FEV1 ≥ 75%, and the ability to perform reproducible spirometry. This study’s exclusion criteria included documented allergic bronchopulmonary aspergillosis (ABPA) confirmed by two positive serum tests or oropharyngeal or expectorated sputum sample with pan-resistant Pseudomonas aeruginosa or Burkholderia cepacia within 12-months before screening. The use of oral corticosteroids ≤ 3-months before baseline testing and clinical instability defined as pulmonary symptoms requiring either oral or intravenous antibiotics ≤ 28-days for a pulmonary exacerbation.

The study protocols Stanford IRB #6218 and Nationwide Children’s IRB #07-00207 CF Biomarkers were reviewed and approved by the Institutional Review Boards at Stanford University Medical Center and Ohio State University School of Medicine. Informed consent and assent were obtained from the parents or the legal guardians of all patients.

After baseline testing, one subject dropped out of the study. The remaining 36 subjects completed the two-year study period. Two-year progression of CF lung disease was evaluated at baseline and three consecutive chest CT scans and lung function testing at times at three, twelve, and 24 months. For each patient, chest CT scans, pulmonary function testing, and infection status were obtained on the same day. Studies were obtained when study subjects were clinically stable and had not received either oral or intravenous antibiotics for a minimum of 28‐days before the study. Of note, this study cohort also is not affected by CFTR potentiator therapy.

## Spirometry

Spirometry was obtained in the standing position for pulmonary function measurements. Pulmonary function measurements (FVC, FEV1, and FEF25–75%) were expressed as percent predicted based on normal prediction equations derived from the Global Lung Function Initiative (GLI-2012) predictive equations for spirometric measurements subsequently generated from the ERS Global Lung function task force (1). This study was initiated prior to the routine use of the lung clearance index for CF clinical studies (2).

## Computed Tomography

Spiral volumetric chest CT was acquired utilizing two different 64-slice scanners (Sensation 64, Siemens Healthineers, Forchheim, Germany, at Lucile Packard Children’s Hospital, and GE VCT, GE Healthcare, Waukesha, WI, at Nationwide Children’s Hospital). Exclusively non-enhanced spirometer-controlled paired inspiratory (100 kVp, 30–50 mAs; Pitch 1.0) and expiratory (100 kVp, 30–50 mAs; Pitch 1.2) CT was routinely performed in a supine position as reported previously (3–5). Inspiratory spiral volumetric CT scans were obtained at ≥ 95% vital capacity (VC), while expiratory spiral volumetric CT scans were obtained near residual volume (RV) at ca. 5 % VC as reported previously (6). The total estimated effective dose for the 4 serial CT scans over the two-year period was 5.4–5.6 mSv. Reconstructions were performed with a medium soft reconstruction kernel (B30f, Standard), as recommended for parenchyma quantification, as well as with a sharp reconstruction kernel (B60f, Bone) (5, 7, 8). All examinations were visually inspected by a reader with more than 20 years of experience (BN) in pediatric chest imaging for adequate inspiration, absence of significant motion artifacts and inclusion of all parts of the chest. The examination protocol and equipment were kept constant during the study period.

## Image assessment

The software YACTA (version 2.8.5.36) segmented lungs and individual lobes fully automatically on inspiratory and expiratory images, as reported previously (8–12). After fully automatic lobe segmentation on inspiratory and expiratory CT datasets, the lobe masks were reviewed by a radiologist. QCT parameters were calculated for the total lung and for each lobe: right upper (RUL), middle (RML), and lower (RLL) lobe, as well as left upper lobe (LUL), lingula (LLi), and left lower lobe (LLL). Total lung volume (TLV) and three different air trapping parameters were calculated for each patient: 1) RVC_856-950_, which is defined as the difference between the expiratory and inspiratory lung volumes with attenuation between -856 and -950 HU divided by the total lung volume without emphysema. The index ranges from -1.0 to 0; higher values (closer to zero) mean more air trapping (13). 2) E/I MLA, defined as the expiratory to the inspiratory ratio of mean lung attenuation with a range from 0 to 1.0; higher values mean more air trapping (13). 3) A1, A2, and A3, which use three thresholds for the definition of air trapping, expressing the size of the defect areas as a fraction of the whole analysed lung parenchyma region. A1 (mild air trapping) represents defects based on liberal criteria, while A3 (severe air trapping) represents defects based on stringent criteria, as published previously (3).

Airways were assessed generation-based in the trachea (G_1_), main stem (G_2_), lobar (G_3_), segmental (G_4_), and aggregated subsegmental bronchi (G_5_ - G_10_). Total diameter (TD), wall thickness (WT), wall percentage (WP), and lumen area (LA) were determined using the parameter-free integral-based method (14). To determine the bronchiectasis index (BEI), the complete bronchial tree is analysed. For every segmented bronchus for which the tapering of the bronchial system is injured an error value is calculated – then the error values of all bronchi are summed up to the bronchiectasis index. Hence, a healthy bronchial tree is assigned a bronchiectasis index of 0, the more bronchiectasis are detected in a bronchial tree, the higher the bronchiectasis index becomes (14). The bronchiectasis index (BEI) was calculated for the total lung, and each lobe (14).

## Statistical analysis

All data were recorded in a dedicated database (Excel®, Microsoft Corp., Redmond, USA), and analyses were performed in R 4.0.2 (15). Data are given as median and interquartile range (Q1 – Q3) due to non-normal distribution. Time-dependent changes in patients demographics, lung volume, air trapping, and airway parameters were assessed by Friedman Tests for continuous and by McNemar Tests for binary variables. The Mann-Whitney-U-Test is used to compare A3 values of patients with infection to patients without infection. Each time point and region is tested separately.

All p-values are of descriptive nature due to the explorative character of the study. Pearson correlation coefficient was calculated for all lobes and time points. The Pearson correlation coefficient was rated as suggested by Karlik for radiological features: 0.0-0.2 as very weak, 0.2-0.4 as weak, 0.4-0.7 as moderate, 0.7-0.9 as strong and 0.9-1.0 as very strong (16). Robust linear and beta mixed models were calculated, where robust methods were used to reduce the impact of outliers. The endpoints (dependent variable) were bronchiectasis index (BE), lumen area (LA5–10), and wall percentage (WP_5–10_). Beta regression was calculated for WP since the dependent variable is a rate and limited by 0 and 1. First, patient and lung region (lobes) were considered as random effect in mixed models. The fixed effects (independent variables) were age, A3, and the examination time point (baseline, 3 months, 12 months, 24 months). Further linear regression models used the difference in BEI, LA_5–10_, or WP_5–10_ between time points as the dependent variable. Independent variables were age, A3, and the first time point considered for the difference. All p-values are of descriptive nature.

Supplemental REFERENCES

1. Quanjer PH, Stanojevic S, Cole TJ, Baur X, Hall GL, Culver B, et al. Multi-ethnic reference values for spirometry for the 3-95 year age range: the global lung function 2012 equations. *European Respiratory Journal* (2012):erj00803-2012. doi:10.1183/09031936.00080312

2. Madanhire T, Ferrand RA, Attia EF, Sibanda EN, Rusakaniko S, Rehman AM. Validation of the global lung initiative 2012 multi-ethnic spirometric reference equations in healthy urban Zimbabwean 7-13 year-old school children: a cross-sectional observational study. *BMC pulmonary medicine* (2020) **20**:56. doi:10.1186/s12890-020-1091-4

3. Goris ML, Zhu HJ, Blankenberg F, Chan F, Robinson TE. An automated approach to quantitative air trapping measurements in mild cystic fibrosis. *Chest* (2003) **123**:1655–63. doi:10.1378/chest.123.5.1655

4. Heussel CP, Kappes J, Hantusch R, Hartlieb S, Weinheimer O, Kauczor H-U, et al. Contrast enhanced CT-scans are not comparable to non-enhanced scans in emphysema quantification. *European Journal of Radiology* (2010) **74**:473–8. doi:10.1016/j.ejrad.2009.03.023

5. Kauczor H-U, Wielpütz MO, Owsijewitsch M, Ley-Zaporozhan J. Computed tomographic imaging of the airways in COPD and asthma. *J Thorac Imaging* (2011) **26**:290–300. doi:10.1097/RTI.0b013e3182277113

6. Robinson TE, Leung AN, Moss RB, Blankenberg FG, al-Dabbagh H, Northway WH. Standardized high-resolution CT of the lung using a spirometer-triggered electron beam CT scanner. *AJR Am J Roentgenol* (1999) **172**:1636–8. doi:10.2214/ajr.172.6.10350305

7. Weinheimer O, Achenbach T, Bletz C, Duber C, Kauczor HU, Heussel CP. About objective 3-d analysis of airway geometry in computerized tomography. *IEEE TRANSACTIONS ON MEDICAL IMAGING* (2008) **27**:64–74. doi:10.1109/TMI.2007.902798

8. Wielpütz MO, Eichinger M, Weinheimer O, Ley S, Mall MA, Wiebel M, et al. Automatic airway analysis on multidetector computed tomography in cystic fibrosis: correlation with pulmonary function testing. *J Thorac Imaging* (2013) **28**:104–13. doi:10.1097/RTI.0b013e3182765785

9. Wielpütz MO, Weinheimer O, Eichinger M, Wiebel M, Biederer J, Kauczor H-U, et al. Pulmonary emphysema in cystic fibrosis detected by densitometry on chest multidetector computed tomography. *PLoS ONE* (2013) **8**:e73142. doi:10.1371/journal.pone.0073142

10. Konietzke P, Wielpütz MO, Wagner WL, Wuennemann F, Kauczor H-U, Heussel CP, et al. Quantitative CT detects progression in COPD patients with severe emphysema in a 3-month interval. *European Radiology* (2020) **30**:2502–12. doi:10.1007/s00330-019-06577-y

11. Konietzke P, Weinheimer O, Wielpütz MO, Wagner WL, Kaukel P, Eberhardt R, et al. Quantitative CT detects changes in airway dimensions and air-trapping after bronchial thermoplasty for severe asthma. *European Journal of Radiology* (2018) **107**:33–8. doi:10.1016/j.ejrad.2018.08.007

12. Konietzke P, Weinheimer O, Wielpütz MO, Savage D, Ziyeh T, Tu C, et al. Validation of automated lobe segmentation on paired inspiratory-expiratory chest CT in 8-14 year-old children with cystic fibrosis. *PLoS ONE* (2018) **13**:e0194557. doi:10.1371/journal.pone.0194557

13. Hersh CP, Washko GR, Estépar RS, Lutz S, Friedman PJ, Han MK, et al. Paired inspiratory-expiratory chest CT scans to assess for small airways disease in COPD. *Respir Res* (2013) **14**:42. doi:10.1186/1465-9921-14-42

14. Weinheimer O, Wielpütz MO, Konietzke P, Heussel CP, Kauczor H-U, Brochhausen C, et al. “Fully automated lobe-based airway taper index calculation in a low dose MDCT CF study over 4 time-points,”. In: Styner MA, Angelini ED, editors. *Medical Imaging 2017: Image Processing*. SPIE (2017). 101330U.

15. Development Core Team R. *R Core Team. R A Language and Environment for Statistical Computing 2014* (2008).

16. Karlik SJ. Exploring and Summarizing Radiologic Data. *American Journal of Roentgenology* (2003) **180**:47–54. doi:10.2214/ajr.180.1.1800047

## Supplementary Figures and Tables

- 1. **Supplementary Figures**


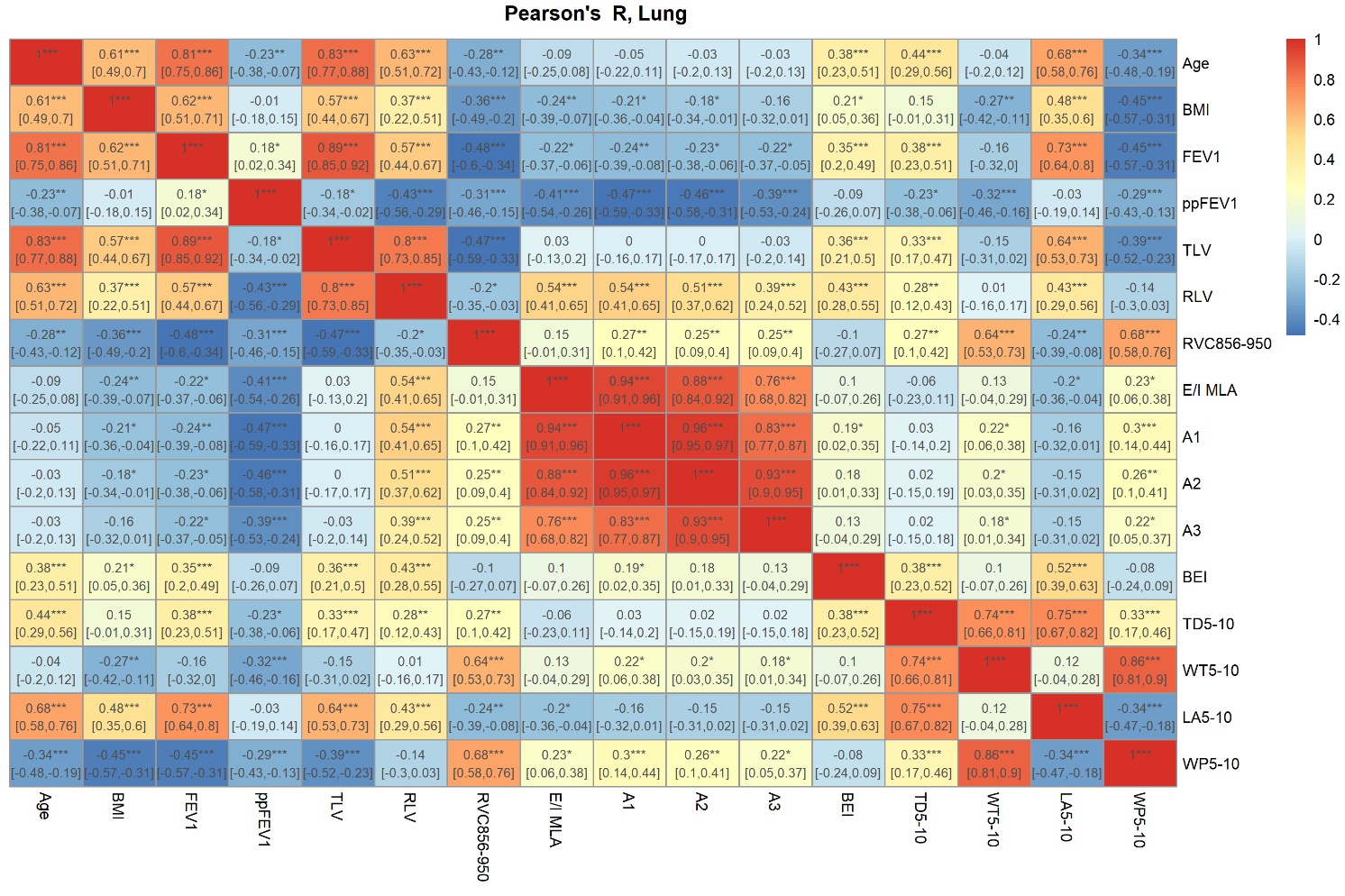
**Supplementary Figure S1A**


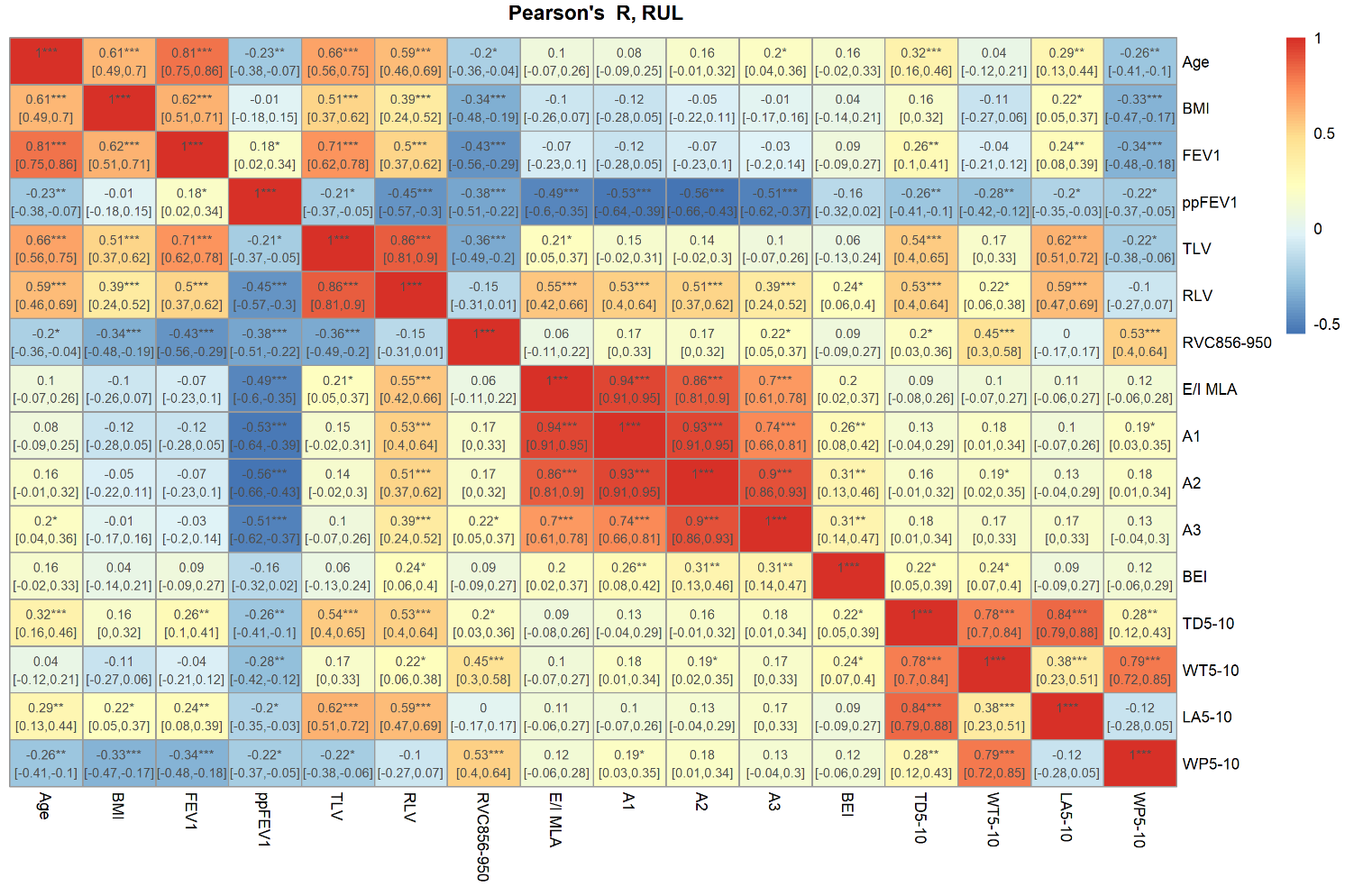
**Supplementary Figure S1B**


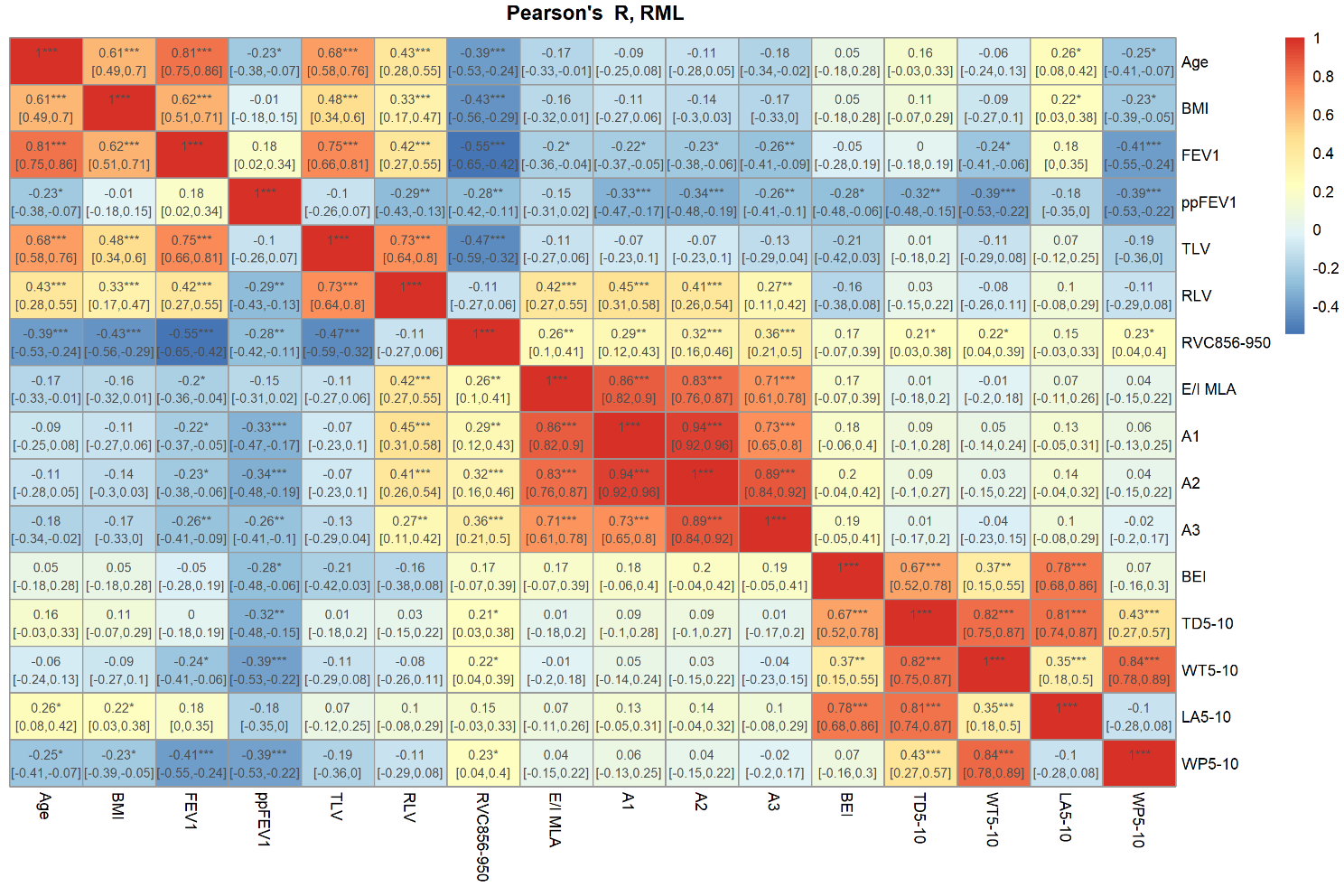
**Supplementary Figure S1C**


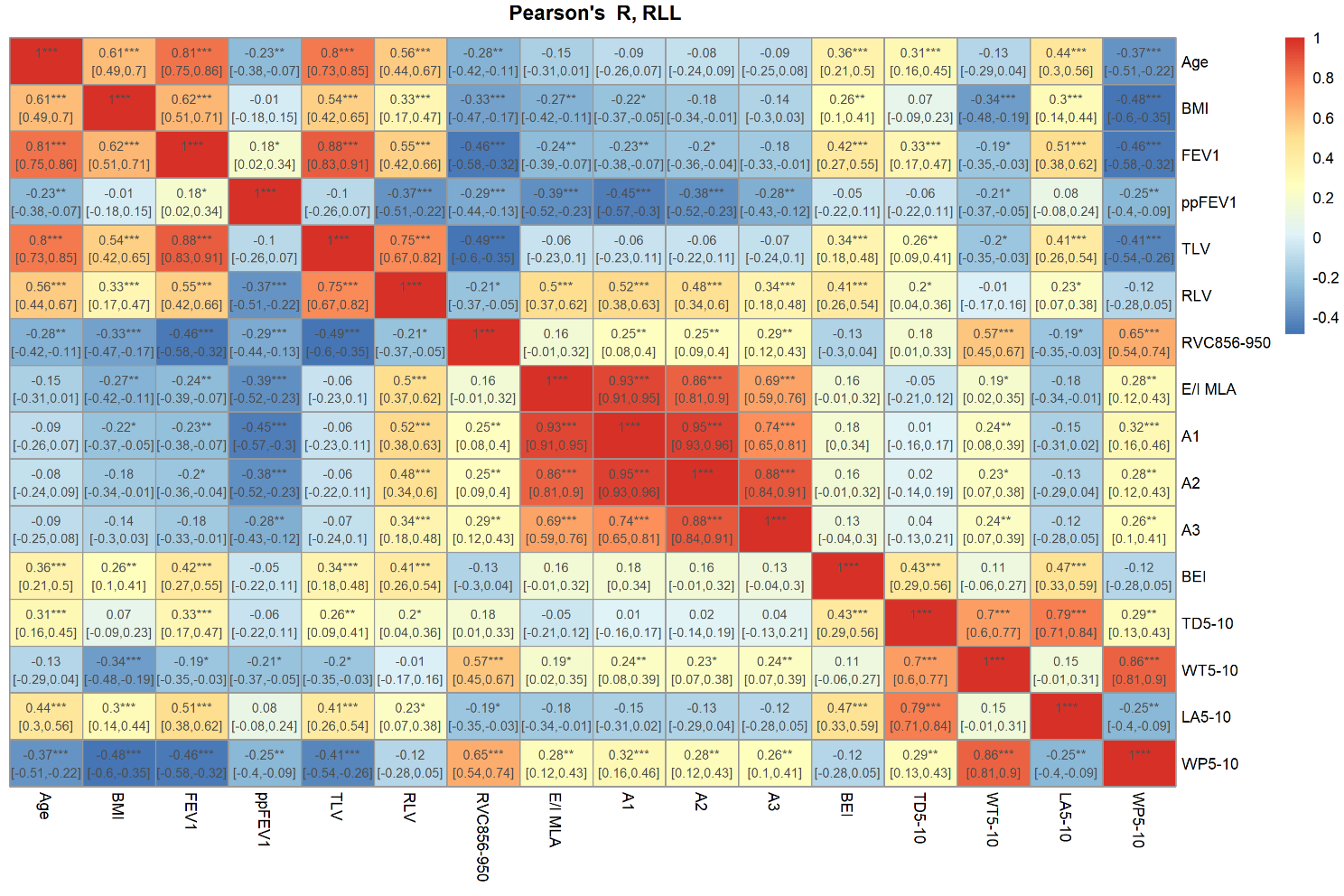
**Supplementary Figure S1D**


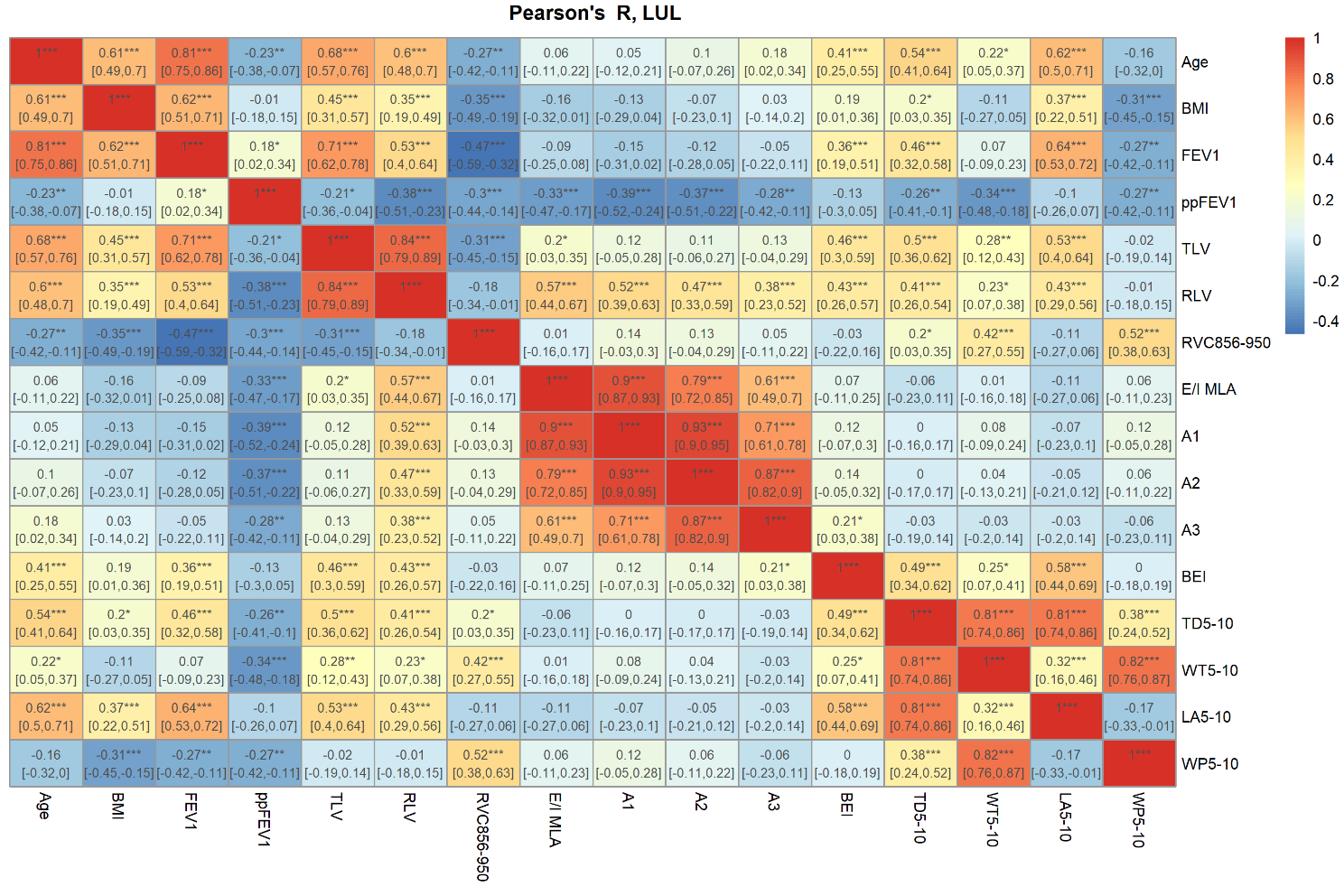
**Supplementary Figure S1E**


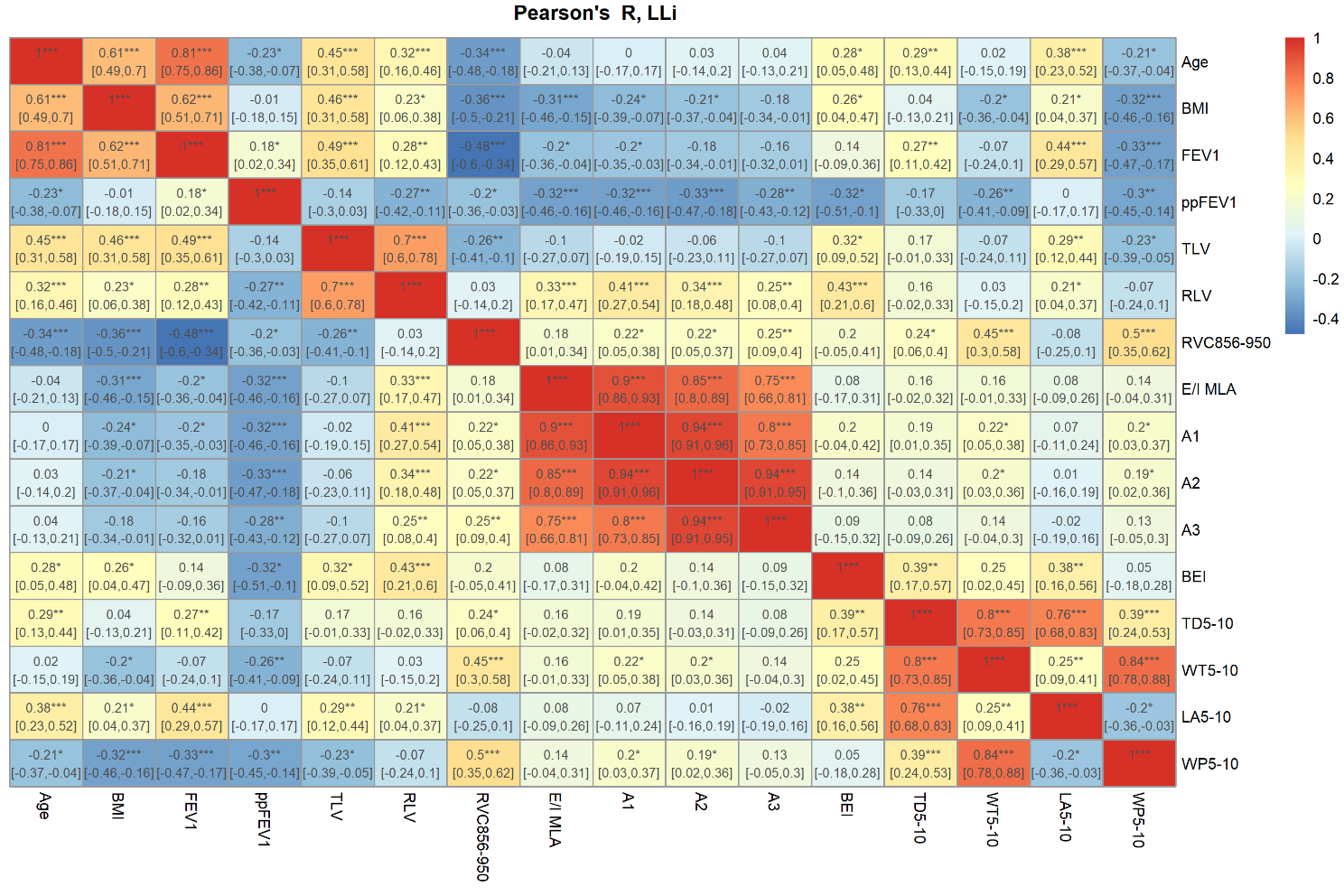
**Supplementary Figure S1F**


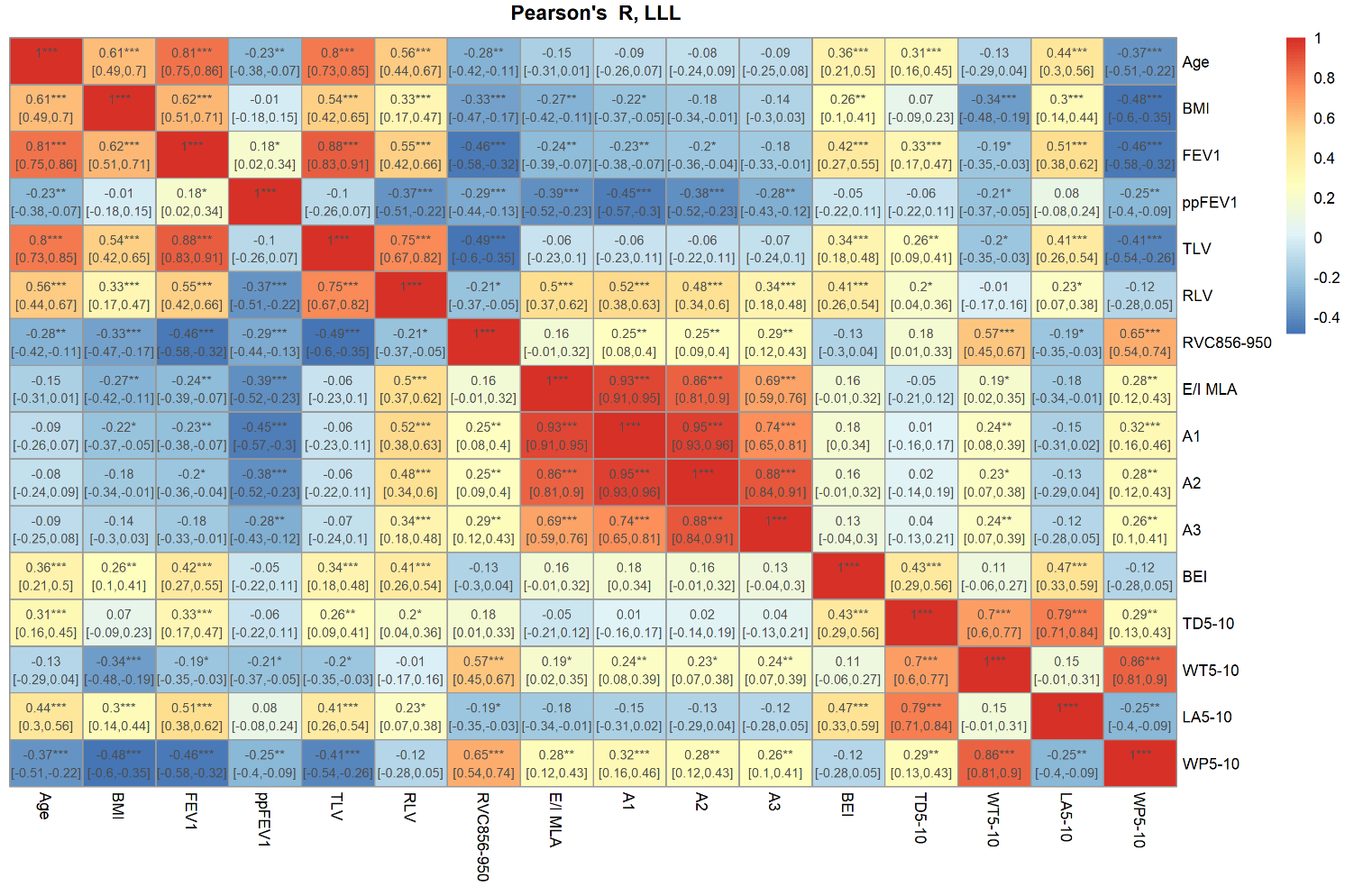


**Supplementary Figure S1G**


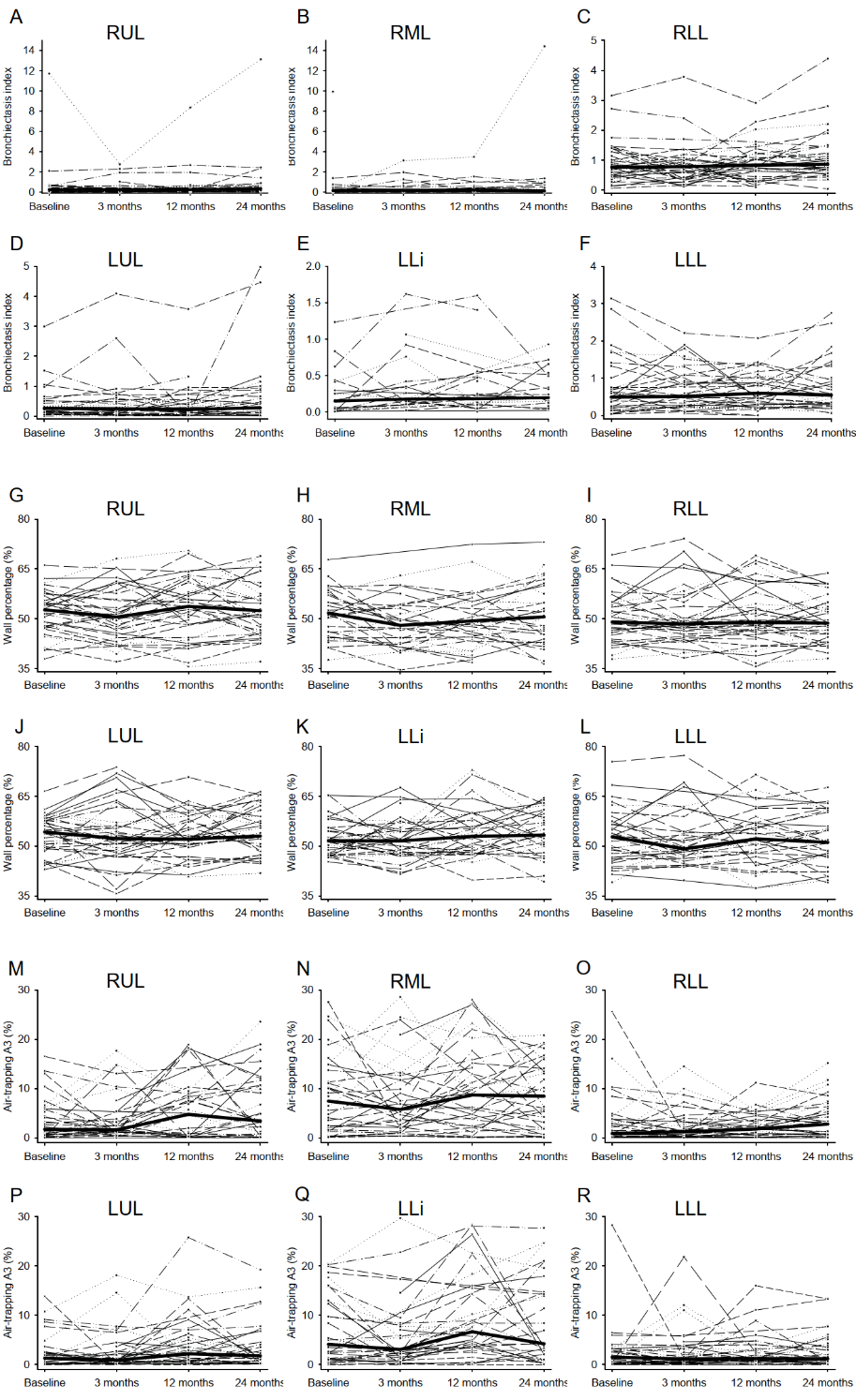


**Supplementary Figure S2.** The airway parameter bronchiectasis index (BEI), wall percentage (WP), and the air trapping (A3) are shown for each lobe: right upper (RUL), middle (RML), and lower (RLL) lobe, as well as left upper lobe (LUL), lingula (LLi), and left lower lobe (LLL). All lobes showed high variability from baseline to 24 months. Over two years BE and A3 tended to increase in all lobes whereas WP5-10 remained stable.


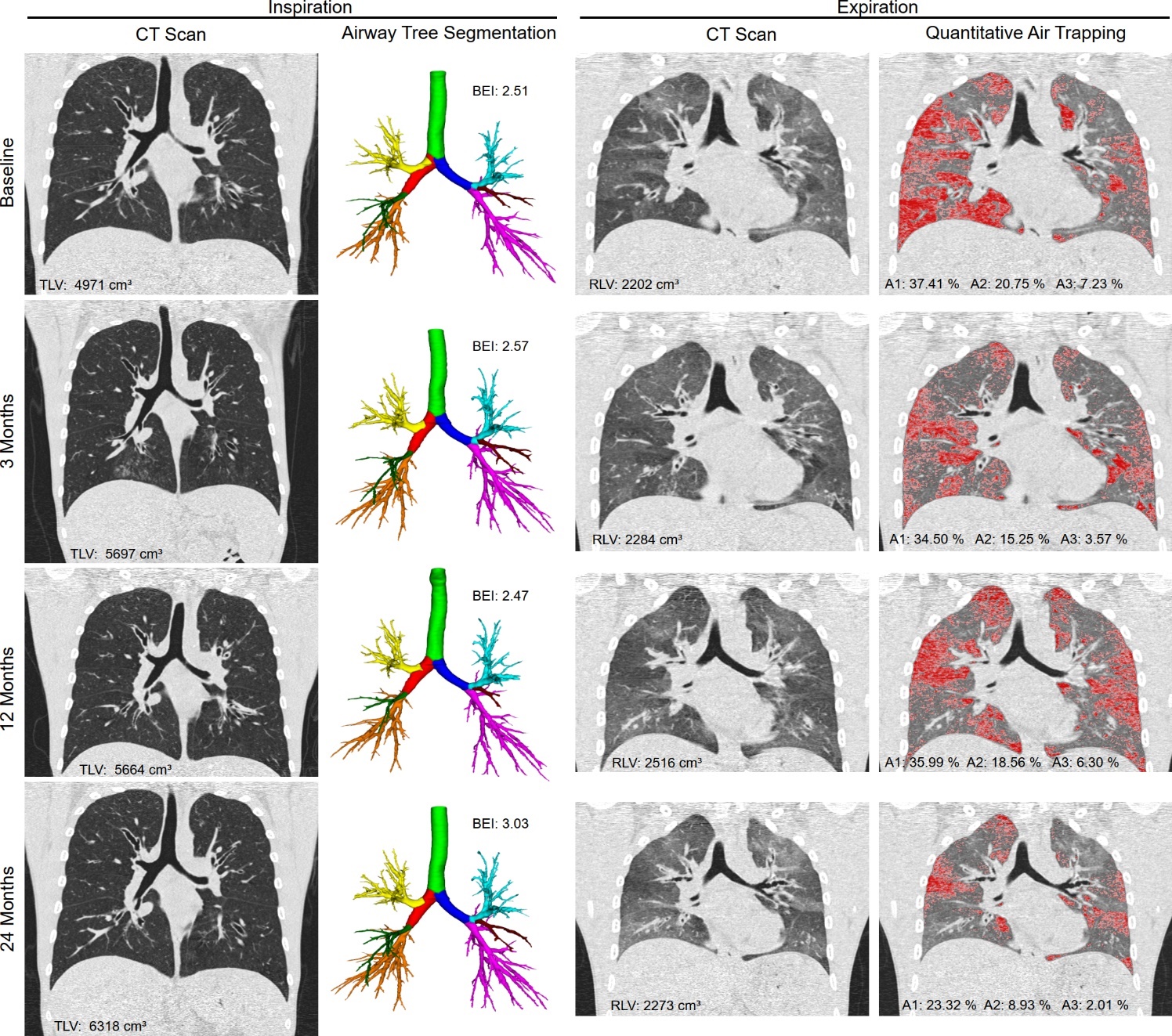


**Supplementary Figure S3.** Representative example of a 15-year-old male cystic fibrosis patient scanned with paired inspiratory-expiratory CT at baseline, and subsequently after 3, 12 and 24 months. The first column shows the inspiratory CT in coronal reconstruction and the second column the segmentation results of the airway tree including labeling of the pulmonary lobes (trachea highlighted in green, right main in red, left main in dark blue, right upper lobe in yellow, middle lobe in green and right lower lobe in orange, left upper lobe in light blue, lingula in red, and left lower lobe in pink). The third column shows the expiratory CT in coronal reconstruction and the fourth column the results of air trapping quantification using the A1 (A1 includes A2 and A3) parameter. The quantitative results TLV, BEI, RLV, A1, A2, A3 are given for each timepoint. The patient showed relatively high quantitative air trapping at baseline, which was stable at the following two scans, but was reduced at 24 months. At the same time BEI was stable at baseline until 12 months but increased at 24 months. Please note, that corresponding ppFEV1 was 80 %, 83 %, 83 % and 92 % respectively.

- 1. **Supplementary Tables**

|  | MRSA | | | PA | | |
| --- | --- | --- | --- | --- | --- | --- |
| Lung | positive | negative | p | positive | negative | p |
| Baseline | | | | | | |
| N | 27 | 9 | 0.827 | 31 | 5 | 0.239 |
| A3 | 2.3  (1.1 – 6.6) | 2.1  (0.9 – 5.2) |  | 2.1  (0.7 – 6) | 6.2  (1.8 – 9.9) |  |
| 3 months | | | | | | |
| N | 31 | 5 | 0.448 | 30 | 6 | 0.058 |
| A3 | 2.3  (1.1 – 3.9) | 3.6  (1.1 – 18.5) |  | 2.8  (1.2 – 6.2) | 1.1  (0.4 – 2.5) |  |
| 12 months | | | | | | |
| N | 30 | 6 | 0.494 | 28 | 6 | 0.297 |
| A3 | 4.3  (1.9 – 7.1) | 3.4  (1.6 – 4.3) |  | 4.1  (1.4 – 5.7) | 5.5  (2.9 – 7.2) |  |
| 24 months | | | | | | |
| N | 28 | 8 | 0.466 | 28 | 8 | 0.676 |
| A3 | 4.2  (1.6 – 7.1) | 6.4  (1.8 – 8.4) |  | 4.3  (1.2 – 7.1) | 5.1  (1.9 – 8.8) |  |

Supplemental Table S1. Differences in air trapping depended on the MRSA and PA infection status. Air trapping (A3) at baseline, 3 months, 12 months, and 24 months in dependence on the infection status for PA (*Pseudomonas aeruginosa)* and MRSA (chronic methicillin-resistant *Staphylococcus aureus*) for the total lung. Data are given as median and interquartile range.

|  | **Baseline** | **3 months** | **12 months** | **24 months** | **p** |
| --- | --- | --- | --- | --- | --- |
| Lung | | | | | |
| BEI | 0.41  (0.28 - 0.7) | 0.39  (0.29 - 0.77) | 0.49  (0.32 - 0.7) | 0.54  (0.36 - 0.88) | 0.156 |
| TD_1_ | 16.9  (15.2 – 18.77) | 16.92  (15.25 – 19.44) | 17.15  (15.53 – 19.7) | 17.72  (16.06 – 20.28) | **<0.001** |
| TD_2_ | 13.15  (11.88 – 14.01) | 13.35  (12.21 – 14.53) | 13.66  (12.43 – 14.5) | 13.78  (12.77 – 15.37) | **<0.001** |
| TD_3_ | 10.8  (10.14 – 11.64) | 10.9  (9.98 – 12.14) | 11.05  (10.17 – 12.02) | 11.14  (9.68 – 12.33) | 0.258 |
| TD_4_ | 8.81  (8.48 – 9.54) | 8.97  (8.02 – 10) | 9.28  (8.35 – 9.95) | 9.25  (8.32 – 10.3) | 0.05 |
| TD_5-10_ | 5.04  (4.63 – 5.46) | 4.96  (4.75 – 5.39) | 5.15  (4.73 – 5.59) | 5.35  (4.82 – 5.78) | **<0.001** |
| WT_1_ | 1.84  (1.7 – 1.96) | 1.85  (1.72 – 2.03) | 1.83  (1.69 – 2.02) | 1.87  (1.74 – 2.03) | 0.387 |
| WT_2_ | 1.73  (1.61 – 2.02) | 1.73  (1.64 – 2.03) | 1.72  (1.61 – 1.98) | 1.7  (1.61 – 2.05) | 0.265 |
| WT_3_ | 1.78  (1.63 – 1.87) | 1.77  (1.63 – 1.91) | 1.8  (1.61 – 1.88) | 1.67  (1.61 – 1.87) | **0.011** |
| WT_4_ | 1.63  (1.5 – 1.73) | 1.67  (1.5 – 1.81) | 1.69  (1.52 – 1.83) | 1.65  (1.51 – 1.78) | 0.463 |
| WT_5-10_ | 0.77  (0.72 – 0.89) | 0.78  (0.69 – 0.91) | 0.84  (0.72 – 0.96) | 0.84  (0.74 – 0.95) | **0.028** |
| LA_1_ | 140.34  (108.38 – 168.66) | 139.92  (106.44 – 188.58) | 147.47  (114.78 – 187.45) | 154.35  (120.23 – 200.89) | ***<*0.001** |
| LA_2_ | 72.29  (60.1 – 84.39) | 76.29  (58.41 – 92.81) | 80.13  (65.26 – 97.62) | 84.46  (69.49 – 106.48) | ***<*0.001** |
| LA_3_ | 42.22  (37.39 – 52.13) | 46.56  (34.8 – 60.75) | 44.56  (35.29 – 55.94) | 46.5  (34.61 – 65.17) | 0.296 |
| LA_4_ | 26.45  (22.81 – 32.35) | 25.53  (18.82 – 35.86) | 26.5  (23.2 – 38.61) | 29.41  (22.69 – 36.78) | **0.013** |
| LA_5-10_ | 9.99  (8.41 – 11.96) | 10.21  (8.11 – 12.08) | 10.9  (8.94 – 11.98) | 11.36  (9.7 – 12.85) | **0.001** |
| WP_1_ | 38.89  (36.34 – 41.37) | 39.74  (36.18 – 43.46) | 38.16  (35.22 – 40.83) | 37.11  (36.15 – 41.1) | **0.015** |
| WP_2_ | 46.94  (44.51 – 48.85) | 46.7  (44.02 – 50.5) | 46.15  (43.54 – 48.78) | 44.57  (42.24 – 48.87) | **<0.001** |
| WP_3_ | 55.04  (51.65 – 58.99) | 54.73  (51.41 – 58.65) | 54.91  (51.35 – 58.97) | 53.27  (49.6 – 58) | **0.033** |
| WP_4_ | 58.72  (55.71 – 61.82) | 59.04  (54.21 – 65.07) | 57.4  (54.56 – 65.17) | 56.6  (55.17 – 62.01) | **0.013** |
| WP_5-10_ | 49.41  (45.48 – 54.65) | 47.69  (46.01 – 52.3) | 50.59  (46.69 – 55.06) | 49.41  (47.17 – 55.81) | 0.248 |
| RUL | | | | | |
| BEI | 0.24  (0.1 – 0.41) | 0.24  (0.1 – 0.41) | 0.25  (0.14 – 0.37) | 0.33  (0.14 – 0.64) | 0.35 |
| TD_3_ | 11.65  (10.56 – 12.46) | 11.97  (10.47 – 13.99) | 12.26  (10.74 – 14.3) | 13.16  (10.27 – 15.36) |  |
| TD_4_ | 8.85  (8 – 10.82) | 8.74  (7.24 – 9.45) | 9.3  (7.95 – 11) | 10.28  (7.62 – 12.44) |  |
| TD_5-10_ | 4.8  (4.49 – 5.62) | 4.7  (4.34 – 5.2) | 5.07  (4.5 – 5.83) | 5.33  (4.83 – 5.97) | **0.038** |
| WT_3_ | 1.71  (1.47 – 1.87) | 1.69  (1.53 – 1.85) | 1.81  (1.55 – 1.93) | 1.69  (1.54 – 1.92) |  |
| WT_4_ | 1.69  (1.44 – 1.94) | 1.67  (1.35 – 1.85) | 1.67  (1.36 – 1.9) | 1.7  (1.46 – 1.9) |  |
| WT_5-10_ | 0.83  (0.68 – 0.96) | 0.79  (0.6 – 0.96) | 0.91  (0.62 – 1.12) | 0.89  (0.74 – 1.05) | **0.048** |
| LA_3_ | 54.98  (44.42 – 66.53) | 57.38  (42.04 – 98.48) | 58.64  (40.1 – 103.58) | 68.15  (38.53 – 118.07) |  |
| LA_4_ | 24.5  (19.01 – 37.57) | 21.33  (14.91 – 27.07) | 25.87  (18.61 – 39.67) | 36.85  (14.67 – 57.67) |  |
| LA_5-10_ | 8.27  (7.38 – 11.92) | 9.05  (7.21 – 11.09) | 8.86  (7.53 – 10.8) | 10.76  (8.24 – 12.28) | **0.029** |
| WP_3_ | 50.7  (44.31 – 54.47) | 48.27  (45.01 – 56.04) | 50.97  (43.09 – 55.35) | 44.85  (36.36 – 54.71) |  |
| WP_4_ | 59.92  (51.34 – 63.54) | 61.52  (53.04 – 68.09) | 57.73  (51.45 – 65.12) | 56.88  (48.59 – 66.99) |  |
| WP_5-10_ | 52.7  (47.75 – 55.81) | 50.5  (45.16 – 55.18) | 53.72  (48.15 – 60.99) | 52.42  (46.07 – 57.33) | 0.121 |
| RML | | | | | |
| BEI | 0.16  (0.08 – 0.39) | 0.18  (0.08 – 0.53) | 0.22  (0.07 – 0.81) | 0.12  (0.06 – 0.49) | 0.172 |
| TD_3_ | 8.75  (7.99 – 9.46) | 8.76  (8.32 – 9.73) | 8.63  (8.17 – 9.58) | 8.97  (8.57 – 9.75) |  |
| TD_4_ | 6.25  (5.08 – 6.81) | 6.55  (6.11 – 7.2) | 6.69  (5.65 – 7.45) | 6.56  (5.72 – 7.96) |  |
| TD_5-10_ | 4.29  (3.85 – 4.5) | 4.21  (3.74 – 4.7) | 4.24  (3.94 – 4.59) | 4.39  (4.01 – 4.9) | 0.331 |
| WT_3_ | 1.64  (1.5 – 1.77) | 1.64  (1.5 – 1.9) | 1.6  (1.43 – 1.77) | 1.63  (1.53 – 1.81) |  |
| WT_4_ | 1.19  (0.85 – 1.44) | 1.41  (1.11 – 1.56) | 1.4  (1.02 – 1.53) | 1.32  (0.99 – 1.59) |  |
| WT_5-10_ | 0.68  (0.54 – 0.81) | 0.62  (0.55 – 0.72) | 0.63  (0.6 – 0.72) | 0.66  (0.58 – 0.82) | 0.528 |
| LA_3_ | 24.09  (19.31 – 28.29) | 24.61  (19.85 – 29.35) | 22.26  (17.53 – 29.52) | 25.76  (19.9 – 28.39) |  |
| LA_4_ | 10.47  (7.87 – 13.56) | 11.71  (8.98 – 13.8) | 10.87  (8.88 – 15.63) | 12.05  (10.4 – 19.23) |  |
| LA_5-10_ | 6.45  (6.02 – 7.82) | 7.26  (5.72 – 8.94) | 7.11  (6.43 – 8.44) | 7.69  (6.46 – 9.06) | 0.301 |
| WP_3_ | 61.15  (57.13 – 64.02) | 60.6  (57.22 – 64.34) | 60.78  (55.04 – 65.23) | 59.55  (56.34 – 63.67) |  |
| WP_4_ | 58.94  (52.12 – 67.56) | 62.42  (56.68 – 67.28) | 61.38  (56.14 – 69.54) | 60.15  (54.71 – 64.05) |  |
| WP_5-10_ | 51.71  (45.01 – 57.79) | 48.07  (44.13 – 53) | 49.35  (43.41 – 55.24) | 50.33  (44.2 – 56.82) | 0.540 |
| RLL | | | | | |
| BEI | 0.77  (0.45 – 1.27) | 0.79  (0.41 – 1.05) | 0.84  (0.59 – 1.11) | 0.86  (0.68 – 1.23) | 0.954 |
| TD_3_ | 11.84  (10.11 – 13.51) | 11.71  (10.3 – 13.76) | 12.35  (10.42 – 13.54) | 11.86  (10.29 – 13.06) |  |
| TD_4_ | 9.38  (8.24 – 10.87) | 9.59  (8.69 – 10.48) | 9.88  (8.44 – 11.08) | 10.14  (9.18 – 11.2) |  |
| TD_5-10_ | 5.3  (4.94 – 5.96) | 5.37  (5.08 – 5.75) | 5.42  (5.12 – 5.84) | 5.67  (5.31 – 6.07) | **0.010** |
| WT_3_ | 1.51  (1.37 – 1.69) | 1.58  (1.39 – 1.91) | 1.6  (1.36 – 1.77) | 1.6  (1.34 – 1.75) |  |
| WT_4_ | 1.55  (1.29 – 1.71) | 1.58  (1.38 – 1.73) | 1.63  (1.44 – 1.84) | 1.71  (1.45 – 1.88) |  |
| WT_5-10_ | 0.85  (0.73 – 0.98) | 0.83  (0.72 – 1.03) | 0.86  (0.74 – 0.99) | 0.89  (0.8 – 1) | 0.145 |
| LA_3_ | 53.98  (43.4 – 78.61) | 54.26  (39.91 – 88.69) | 58.27  (46.44 – 76.56) | 58.03  (40.99 – 74.48) |  |
| LA_4_ | 33.75  (22.33 – 43.21) | 30.17  (24.39 – 41.79) | 35.28  (23.95 – 44.33) | 37.57  (23.41 – 44.98) |  |
| LA_5-10_ | 12.03  (9.18 – 14.46) | 11.71  (9.3 – 13.3) | 11.61  (10.23 – 13.8) | 13.2  (10.69 – 14.36) | 0.131 |
| WP_3_ | 46.59  (41.82 – 49.83) | 49.88  (44.25 – 53.34) | 45.53  (42.24 – 50.22) | 45.45  (42.13 – 52.16) |  |
| WP_4_ | 54.18  (51.43 – 59.27) | 56.76  (49.71 – 60.01) | 56.1  (49.86 – 61.75) | 53.55  (48.05 – 58.95) |  |
| WP_5-10_ | 49  (44.77 – 54.74) | 48.45  (45.17 – 54.07) | 48.86  (45.81 – 55.32) | 48.69  (45.56 – 54.26) | 0.530 |
| LUL | | | | | |
| BEI | 0.26  (0.14 – 0.47) | 0.25  (0.13 – 0.6) | 0.23  (0.09 – 0.55) | 0.28  (0.1 – 0.82) | 0.753 |
| TD_3_ | 11.69  (10.8 – 12.83) | 11.7  (10.49 – 13.1) | 11.5  (10.92 – 12.86) | 12.18  (10.79 – 13.05) |  |
| TD_4_ | 10.33  (9.58 – 11.41) | 10.72  (9.18 – 12.27) | 10.84  (9.57 – 11.71) | 10.47  (8.86 – 12.17) |  |
| TD_5-10_ | 5  (4.58 – 5.54) | 5.42  (4.8 – 5.78) | 5.35  (4.85 – 5.73) | 5.32  (4.91 – 6.28) | **0.012** |
| WT_3_ | 4.58  (1.7 – 2.12) | 4.8  (1.67 – 2.25) | 4.85  (1.62 – 2.19) | 4.91  (1.64 – 2.36) |  |
| WT_4_ | 1.95  (1.8 – 2.3) | 1.92  (1.78 – 2.16) | 1.91  (1.69 – 2.29) | 1.88  (1.7 – 2.05) |  |
| WT_5-10_ | 0.89  (0.77 – 0.97) | 0.88  (0.76 – 1.09) | 0.9  (0.77 – 1.03) | 0.91  (0.8 – 1.15) | 0.116 |
| LA_3_ | 48.38  (38.54 – 55.85) | 50.2  (40.03 – 59.38) | 48.99  (37.8 – 61.21) | 56.08  (38.42 – 67.61) |  |
| LA_4_ | 32.71  (21.6 – 42.09) | 35.51  (22.79 – 46.09) | 33.96  (24.66 – 41.26) | 31.64  (22.56 – 41.49) |  |
| LA_5-10_ | 9.14  (7.23 – 10.9) | 9.47  (8.38 – 11.7) | 10.74  (8.71 – 12.24) | 10.19  (8.72 – 12.35) | **0.002** |
| WP_3_ | 54.71  (49.18 – 61.71) | 53.97  (49.22 – 61.31) | 54.95  (48.13 – 61.31) | 54.68  (47.31 – 60.36) |  |
| WP_4_ | 63.03  (56.33 – 68.13) | 63.14  (55.88 – 69.72) | 62.12  (55.74 – 66.52) | 61.59  (56.22 – 67.9) |  |
| WP_5-10_ | 54.24  (49.33 – 57.68) | 52.27  (48.47 – 56.45) | 52.1  (50.01 – 58.31) | 53.04  (49.19 – 59.87) | 0.557 |
| LLi | | | | | |
| BEI | 0.15  (0.02 – 0.32) | 0.18  (0.11 – 0.42) | 0.18  (0.1 – 0.47) | 0.20  (0.14 – 0.5) | 0.934 |
| TD_4_ | 8.63  (8.06 – 9.28) | 8.96  (7.66 – 9.74) | 8.82  (7.93 – 10.03) | 8.79  (8.17 – 9.68) |  |
| TD_5-10_ | 4.78  (4.3 – 5.21) | 5  (4.26 – 5.45) | 5.01  (4.38 – 5.55) | 5.25  (4.72 – 5.72) | **0.002** |
| WT_4_ | 1.53  (1.41 – 1.9) | 1.62  (1.44 – 1.79) | 1.66  (1.4 – 1.82) | 1.63  (1.48 – 1.78) |  |
| WT_5-10_ | 0.74  (0.65 – 0.92) | 0.8  (0.72 – 0.92) | 0.85  (0.68 – 1) | 0.9  (0.76 – 1.04) | 0.061 |
| LA_4_ | 23.84  (18.25 – 26.18) | 25.89  (17.83 – 32.66) | 22.66  (18.81 – 30.09) | 22.48  (18.14 – 30.59) |  |
| LA_5-10_ | 8.09  (7.22 – 9.87) | 9.2  (7.05 – 11.18) | 8.39  (7.42 – 10.04) | 9.47  (7.48 – 11.07) | **0.021** |
| WP_4_ | 59.71  (55.04 – 65.25) | 57.27  (54.2 – 63.33) | 59.43  (54.65 – 64.96) | 57.68  (54.46 – 65.44) |  |
| WP_5-10_ | 51.61  (47.74 – 55.53) | 51.59  (47.85 – 55.46) | 52.94  (48.62 – 58.23) | 53.31  (49.94 – 60.19) | 0.220 |
| LLL | | | | | |
| BEI | 0.49  (0.03 – 0.37) | 0.51  (0.03 – 0.46) | 0.6  (0.02 – 0.51) | 0.55  (0.04 – 0.6) | 0.813 |
| TD_3_ | 12.46  (11.63 – 13.69) | 12.14  (11.48 – 14.19) | 12.71  (11.55 – 13.88) | 12.79  (11.38 – 14) |  |
| TD_4_ | 10.36  (9.61 – 11.31) | 10.74  (9.46 – 11.54) | 10.78  (9.62 – 11.36) | 10.43  (9.67 – 11.63) |  |
| TD_5-10_ | 5.53  (4.99 – 6.01) | 5.54  (5.06 – 6.41) | 5.67  (5.04 – 6.42) | 5.87  (5.25 – 6.48) | **0.006** |
| WT_3_ | 1.98  (1.87 – 2.23) | 1.99  (1.83 – 2.18) | 1.95  (1.79 – 2.25) | 1.87  (1.81 – 2.24) |  |
| WT_4_ | 1.77  (1.68 – 1.98) | 1.83  (1.64 – 2) | 1.8  (1.69 – 2.11) | 1.78  (1.67 – 1.94) |  |
| WT_5-10_ | 0.89  (0.78 – 1.14) | 0.93  (0.78 – 1.05) | 0.97  (0.79 – 1.15) | 0.96  (0.78 – 1.14) | 0.218 |
| LA_3_ | 58.64  (47.26 – 66.54) | 57.06  (46.02 – 77.02) | 58.73  (50.04 – 72.48) | 63.79  (48.09 – 76.93) |  |
| LA_4_ | 34.24  (27.35 – 40.74) | 37.83  (29.31 – 45.53) | 34.45  (29.39 – 40.97) | 38.53  (31.81 – 48.18) |  |
| LA_5-10_ | 11.44  (9.52 – 13.19) | 12.32  (9.25 – 15.37) | 12.42  (10.3 – 14.92) | 13.5  (11.08 – 16.05) | 0.095 |
| WP_3_ | 53.62  (51.27 – 58.31) | 52.11  (49.13 – 58.42) | 52.38  (48.06 – 57.09) | 51.67  (47.53 – 56.99) |  |
| WP_4_ | 58.68  (54.01 – 61.12) | 57.36  (51.78 – 60.86) | 57.52  (54.44 – 62.98) | 55.87  (52.54 – 60.53) |  |
| WP_5-10_ | 52.76  (46.48 – 56.87) | 49.17  (46.87 – 54.2) | 52.17  (47.94 – 56.91) | 51.13  (47.49 – 60.25) | 0.851 |

Supplemental Table S2. Lobe-based temporal evolution of airway parameters in the lung. Bronchiectasis index (BEI), total diameter (TD), wall thickness (WT), lumen area (LA), wall percentage (WP) were calculated for the right upper (RUL), middle (RML) and lower lobe (RLL), the left upper lobe (LUL), the lingula (LLi) and the left lower lobe (LLL) at baseline, 3 months, 12 months, and 24 months. Results are also shown for the individual airway generations (3^rd^, 4^th^_,_ and 5^th^-10^th^). Data are given as median and interquartile range.

|  | **Baseline** | **3 months** | **12 months** | **24 months** | **p** |
| --- | --- | --- | --- | --- | --- |
| RUL | | | | | |
| Volume | 643.6  (549.65 – 745.69) | 656.12  (525.61 – 765.4) | 689.77  (552.6 – 822.32) | 757.31  (606.69 – 973.99) | ***<*0.001** |
| Residual Vol. | 251.3 (171.8 -–285.1) | 243.2  (183.7 - 316.3) | 275.02  (197.37 - 338.95) | 280.56  (212.62 - 382.42) | ***<*0.001** |
| E/I MLA | 0.67  (0.62 – 0.74) | 0.69  (0.63 – 0.75) | 0.7  (0.65 – 0.74) | 0.7  (0.62 – 0.76) | 0.814 |
| RVC_856-950_ | -0.62  (-0.69 – -0.53) | -0.62  (-0.68 – -0.52) | -0.61  (-0.71 – -0.49) | -0.64  (-0.7 – -0.51) | 0.879 |
| A1 | 24.11  (8.69 – 43.88) | 28.91  (10.51 – 49.81) | 29.97  (12.98 – 52.41) | 36.16  (9.54 – 49.96) | 0.416 |
| A2 | 8.75  (2.87 – 19.36) | 10.44  (3.43 – 18.59) | 13.97  (2.9 – 26.99) | 15.53  (3.95 – 28.77) | 0.325 |
| A3 | 1.72  (0.23 – 5.84) | 1.62  (0.84 – 5.3) | 4.76  (0.24 – 8.67) | 3.4  (0.61 – 10.62) | 0.198 |
| RML | | | | | |
| Volume | 306.17  (237.53 – 344.09) | 263.83  (230.63 – 338.12) | 316.7  (259.49 – 376.93) | 321.7  (272.02 – 382.38) | ***<*0.001** |
| Residual Vol. | 118.69  (78.48 - 157.70) | 101.67  (84.24 - 144.36) | 131.31  (97.34 - 167.90) | 132.315  (80.880 - 171.770) | 0.122 |
| E/I MLA | 0.76  (0.69 – 0.78) | 0.75  (0.71 – 0.81) | 0.76  (0.71 – 0.79) | 0.75  (0.69 – 0.8) | 0.776 |
| RVC_856-950_ | -0.67  (-0.71 – -0.57) | -0.68  (-0.72 – -0.58) | -0.67  (-0.73 – -0.6) | -0.69  (-0.73 – -0.62) | 0.832 |
| A1 | 53.28  (32.96 – 61.84) | 53.72  (35.14 – 69) | 57.73  (41.22 – 68.81) | 49.78  (25.96 – 67.63) | 0.274 |
| A2 | 28.19  (10.99 – 37.95) | 26.35  (13 – 43.58) | 27.92  (18.2 – 46.02) | 27.41  (11.93 – 46.61) | 0.298 |
| A3 | 7.45  (1.71 – 14.56) | 5.74  (2.53 – 12.78) | 8.69  (2.7 – 15.67) | 8.46  (1.93 – 14.08) | 0.687 |
| RLL | | | | | |
| Volume | 1023.27  (795.62 – 1208.09) | 1069.53  (821.59 – 1250.5) | 1104.62  (885.16 – 1321.49) | 1183.42  (906.7 – 1421.05) | ***<*0.001** |
| Residual Vol. | 303.1  (210.4 - 411.9) | 315.8  (232.1 - 420.4) | 330.2  (255.3 - 452.9) | 343.4  (256.6 - 507.2) | 0.001 |
| E/I MLA | 0.63  (0.55 – 0.7) | 0.64  (0.59 – 0.7) | 0.63  (0.61 – 0.68) | 0.63  (0.58 – 0.72) | 0.842 |
| RVC_856-950_ | -0.64  (-0.69 – -0.47) | -0.6  (-0.69 – -0.54) | -0.63  (-0.72 – -0.49) | -0.63  (-0.71 – -0.52) | 0.804 |
| A1 | 12.59  (6.39 – 33.87) | 17.43  (9.52 – 35.82) | 20.25  (12 – 28.87) | 17.66  (8.91 – 38.43) | 0.954 |
| A2 | 4.03  (1.45 – 15.32) | 6.97  (2.01 – 16.48) | 7.78  (3.34 – 12.67) | 9.18  (2.44 – 20.94) | 0.241 |
| A3 | 0.88  (0.17 – 3.77) | 1.23  (0.3 – 3.27) | 1.8  (0.57 – 3.3) | 2.79  (0.62 – 5.8) | 0.211 |
| LUL | | | | | |
| Volume | 493.67  (428.77 – 614.99) | 558.26  (446.62 – 635.21) | 563.86  (475.51 – 685.01) | 628.54  (565.11 – 773.16) | ***<*0.001** |
| Residual Vol. | 156.31  (111.00 - 238.95) | 170.38  (127.17 - 260.15) | 179.3  (142.1 - 268.7) | 196.32  (146.60 - 298.87) | ***<*0.001** |
| E/I MLA | 0.63  (0.55 – 0.7) | 0.66  (0.59 – 0.7) | 0.66  (0.6 – 0.71) | 0.65  (0.59 – 0.72) | 0.310 |
| RVC_856-950_ | -0.62  (-0.71 – -0.55) | -0.64  (-0.69 – -0.54) | -0.61  (-0.71 – -0.49) | -0.66  (-0.71 – -0.56) | 0.649 |
| A1 | 13.18  (4.57 – 31.83) | 16.55  (8.75 – 32.11) | 20.42  (12.52 – 38.75) | 21.89  (7.5 – 38.83) | 0.087 |
| A2 | 5.22  (1.1 – 11.71) | 4.82  (1.46 – 11.2) | 10.57  (2.72 – 19.72) | 6.44  (2.91 – 15.82) | 0.068 |
| A3 | 1.27  (0.15 – 2.55) | 0.85  (0.14 – 2.95) | 2.17  (0.49 – 5.98) | 1.74  (0.44 – 4.37) | **0.025** |
| LLi | | | | | |
| Volume | 238.46  (187.71 – 281.98) | 249.66  (173.65 – 329.15) | 250.05  (197.52 – 342.72) | 258.69  (166.74 – 378.13) | ***<*0.001** |
| Residual Vol. | 83.77  (48.08 - 127.32) | 76.03  (50.67 - 120.82) | 90.39  (69.58 - 140.68) | 89.50  (61.98 - 140.72) | 0.1524 |
| E/I MLA | 0.71  (0.62 – 0.78) | 0.69  (0.64 – 0.77) | 0.73  (0.66 – 0.79) | 0.71  (0.62 – 0.77) | 0.329 |
| RVC_856-950_ | -0.63  (-0.67 – -0.53) | -0.65  (-0.68 – -0.53) | -0.63  (-0.68 – -0.52) | -0.64  (-0.69 – -0.57) | 0.440 |
| A1 | 34.6  (14.73 – 52.88) | 36.48  (13.73 – 47.51) | 47.49  (21.67 – 59) | 35.64  (17.52 – 56.47) | 0.081 |
| A2 | 14.7  (4.14 – 31.12) | 12.71  (5.93 – 24.78) | 23.66  (9.41 – 35.6) | 17.34  (7.86 – 38.03) | 0.245 |
| A3 | 4.08  (1.22 – 12.94) | 3.04  (1.35 – 8.2) | 6.64  (3.48 – 15.24) | 4.19  (2.04 – 14.63) | 0.180 |
| LLL | | | | | |
| Volume | 974.49  (796.02 – 1159.57) | 1029.78  (758.6 – 1138.62) | 1069.36  (777.11 – 1242.27) | 1142.26  (789.6 – 1392.51) | ***<*0.001** |
| Residual Vol. | 273.9  (188.2 - 355.7) | 248.11  (195.55 - 346.51) | 319.4  (222.3 - 378.4) | 329.33  (244.23 - 466.99) | ***<*0.001** |
| E/I MLA | 0.59  (0.56 – 0.64) | 0.6  (0.57 – 0.67) | 0.6  (0.58 – 0.65) | 0.61  (0.55 – 0.66) | 0.888 |
| RVC_856-950_ | -0.62  (-0.68 – -0.5) | -0.59  (-0.69 – -0.5) | -0.59  (-0.71 – -0.46) | -0.61  (-0.7 – -0.49) | 0.649 |
| A1 | 12.23  (5.66 – 21.15) | 13.73  (6.39 – 28.22) | 15.89  (8.06 – 22.84) | 13.61  (5.32 – 23.81) | 0.649 |
| A2 | 4.94  (1.67 – 8.83) | 5.95  (1.9 – 12.22) | 5.3  (2.68 – 9.79) | 4.22  (2.13 – 11.16) | 0.623 |
| A3 | 1.48  (0.29 – 3.14) | 1.12  (0.47 – 3.5) | 1.2  (0.63 – 3.04) | 1.17  (0.66 – 3.74) | 0.435 |

**Supplemental Table S3. Lobe-based temporal evolution of volume and air trapping parameters.** Lobe volume and air trapping parameters (RVC_856-950_, E/I MLA, A1-3) were calculated for the right upper (RUL), middle (RML) and lower lobe (RLL), the left upper lobe (LUL), the lingula (LLi) and the left lower lobe (LLL) at baseline, 3 months, 12 months, and 24 months. Data are given as median and interquartile range.

|  | **Est CI (LL – UL)** | **SE** | | **p-value** |
| --- | --- | --- | --- | --- |
| BEI | | | | |
| Baseline and 3 months | | | | |
| Age | 0.043 (0.014 - 0.072) | 0.015 | | **0.007** |
| A3 | 0.017 (0.007 - 0.028) | 0.001 | | **0.003** |
| Baseline | 0.403 (0.351 - 0.455) | 0.027 | | **<0.001** |
| Baseline and 12 months | | | | |
| Age | 0.003 (-0.025 - 0.019) | 0.011 | | 0.791 |
| A3 | 0.007 (-0.004 - 0.017) | 0.005 | | 0.215 |
| Baseline | 0.696 (0.643 - 0.973) | 0.031 | | **<0.001** |
| Baseline and 24 months | | | | |
| Age | 0.230 (-0.002 - 0.048) | 0.013 | | 0.086 |
| A3 | 0.008 (-0.001 - 0.013) | 0.005 | | 0.091 |
| Baseline | 0.983 (0.925 - 1.041) | 0.030 | | **<0.001** |
| 3 months and 12 months | | | | |
| Age | 0.006 (-0.022 - 0.034) | 0.014 | | 0.676 |
| A3 | 0.011 (0.001 - 0.021) | 0.005 | | 0.058 |
| 3 months | 0.764 (0.668 - 0.861) | 0.026 | | **<0.001** |
| 3 months and 24 months | | | | |
| Age | 0.022 (-0.009 - 0.052) | 0.016 | | 0.175 |
| A3 | 0.005 (-0.005 - 0.016) | 0.005 | | 0.311 |
| 3 months | 0.957 (0.849 - 1.066) | 0.055 | | **<0.001** |
| LA_5-10_ | | | | |
| Baseline and 3 months | | | | |
| Age | 0.357 (0.139 - 0.575) | | 0.111 | **0.003** |
| A3 | 0.023 (-0.031 - 0.077) | | 0.028 | 0.413 |
| Baseline | 0.378 (0.285 - 0.471) | | 0.048 | **<0.001** |
| Baseline and 12 months | | | | |
| Age | 0.185 (0.03 - 0.341) | 0.079 | | **0.026** |
| A3 | -0.031 (-0.078 - 0.015) | 0.024 | | 0.197 |
| Baseline | 0.489 (0.403 - 0.575) | 0.044 | | **<0.001** |
| Baseline and 24 months | | | | |
| Age | 0.294 (0.07 - 0.519) | 0.115 | | **0.015** |
| A3 | 0.043 (-0.023 - 0.11) | 0.034 | | 0.213 |
| Baseline | 0.466 (0.355 - 0.578) | 0.057 | | **<0.001** |
| 3 months and 12 months | | | | |
| Age | 0.294 (0.135 - 0.454) | 0.081 | | **0.001** |
| A3 | 0.006 (-0.042 - 0.053) | 0.024 | | 0.815 |
| 3 months | 0.412 (0.32 - 0.504) | 0.047 | | **<0.001** |
| 3 months and 24months | | | | |
| Age | 0.293 (0.132 - 0.454) | 0.082 | | **0.001** |
| A3 | 0.026 (-0.028 - 0.079) | 0.027 | | 0.356 |
| 3 months | 0.552 (0.443 - 0.66) | 0.055 | | **<0.001** |
| WP_5-10_ | | | | |
| Baseline and 3 months | | | | |
| Age | -0.782 (-1.332 - -0.233) | 0.28 | | **0.009** |
| A3 | 0.013 (-0.088 - 0.114) | 0.052 | | 0.796 |
| Baseline | 0.404 (0.292 - 0.517) | 0.057 | | **<0.001** |
| Baseline and 12 months | | | | |
| Age | -0.078 (-0.702 - 0.546) | 0.318 | | 0.808 |
| A3 | 0.133 (0.02 - 0.246) | 0.058 | | **0.028** |
| Baseline | 0.617 (0.482 - 0.751) | 0.069 | | **<0.001** |
| Baseline and 24 months | | | | |
| Age | -0.992 (-1.466 - -0.519) | 0.242 | | **<0.001** |
| A3 | 0.1 (-0.021 - 0.221) | 0.062 | | 0.114 |
| Baseline | 0.386 (0.261 - 0.511) | 0.064 | | **<0.001** |
| 3 months and 12 months | | | | |
| Age | 0.225 (-0.482 - 0.932) | 0.361 | | 0.537 |
| A3 | 0.054 (-0.052 - 0.161) | 0.054 | | 0.323 |
| 3 months | 0.546 (0.421 - 0.671) | 0.064 | | **<0.001** |
| 3 months and 24 months | | | | |
| Age | -0.865 (-1.419 - -0.312) | 0.282 | | **0.004** |
| A3 | 0.021 (-0.092 - 0.135) | 0.058 | | 0.716 |
| 3 months | 0.35 (0.214 - 0.485) | 0.069 | | **<0.001** |

Supplemental Table S4. Robust linear mixed model for air trapping at different time points. Robust linear model for all lung regions. Patient and lung region are considered as random effects. Fixed effects are the different time points (baseline, 3 months, 12 months, and 24 months). Endpoints are BE, LA_5-10_ and WP_5-10_. Est = Regression coefficients describes the influence of a fixed effect on the endpoint parameters with the given Cl (LL – UL) = Confidence Interval (Lower Limit – Upper Limit). SE = Standard Error.
